# An Efficient Microarray-Based Genotyping Platform for the Identification of Drug-Resistance Mutations in Majority and Minority Subpopulations of HIV-1 Quasispecies

**DOI:** 10.1371/journal.pone.0166902

**Published:** 2016-12-13

**Authors:** Verónica Martín, Celia Perales, María Fernández-Algar, Helena G. Dos Santos, Patricia Garrido, María Pernas, Víctor Parro, Miguel Moreno, Javier García-Pérez, José Alcamí, José Luis Torán, David Abia, Esteban Domingo, Carlos Briones

**Affiliations:** 1 Centro de Biología Molecular ‘Severo Ochoa’ (CBMSO, CSIC-UAM). Campus de Cantoblanco, Madrid, Spain; 2 Centro de Investigación Biomédica en Red de enfermedades hepáticas y digestivas (CIBERehd), Spain; 3 Liver Unit, Internal Medicine, Laboratory of Malalties Hepàtiques, Vall d’Hebron Institut de Recerca-Hospital Universitari Vall d´Hebron (VHIR-HUVH), Universitat Autònoma de Barcelona. Barcelona, Spain; 4 Department of Molecular Evolution, Centro de Astrobiología (CAB, CSIC-INTA). Torrejón de Ardoz, Madrid, Spain; 5 Biotherapix, SLU. Parque Tecnológico de Madrid, Tres Cantos, Madrid. Spain; 6 AIDS Immunopathogenesis Unit, Instituto de Salud Carlos III. Majadahonda, Madrid, Spain; National and Kapodistrian University of Athens, GREECE

## Abstract

The response of human immunodeficiency virus type 1 (HIV-1) quasispecies to antiretroviral therapy is influenced by the ensemble of mutants that composes the evolving population. Low-abundance subpopulations within HIV-1 quasispecies may determine the viral response to the administered drug combinations. However, routine sequencing assays available to clinical laboratories do not recognize HIV-1 minority variants representing less than 25% of the population. Although several alternative and more sensitive genotyping techniques have been developed, including next-generation sequencing (NGS) methods, they are usually very time consuming, expensive and require highly trained personnel, thus becoming unrealistic approaches in daily clinical practice. Here we describe the development and testing of a HIV-1 genotyping DNA microarray that detects and quantifies, in majority and minority viral subpopulations, relevant mutations and amino acid insertions in 42 codons of the *pol* gene associated with drug- and multidrug-resistance to protease (PR) and reverse transcriptase (RT) inhibitors. A customized bioinformatics protocol has been implemented to analyze the microarray hybridization data by including a new normalization procedure and a stepwise filtering algorithm, which resulted in the highly accurate (96.33%) detection of positive/negative signals. This microarray has been tested with 57 subtype B HIV-1 clinical samples extracted from multi-treated patients, showing an overall identification of 95.53% and 89.24% of the queried PR and RT codons, respectively, and enough sensitivity to detect minority subpopulations representing as low as 5–10% of the total quasispecies. The developed genotyping platform represents an efficient diagnostic and prognostic tool useful to personalize antiviral treatments in clinical practice.

## Introduction

RNA viruses replicate with very high mutation rates, in the range of 10^*−*3^ to 10^*−*5^ substitutions per nucleotide copied, as a result of the absent or low proofreading activity of viral RNA-dependent or DNA-dependent RNA polymerases (the latter also called reverse transcriptases, RTs) [1,2]. Such mutation rates (together with a high frequency of recombination in most viruses and high replication rates) make RNA viruses to evolve as complex, highly heterogeneous and dynamic populations of related but non-identical genomes, termed viral quasispecies [3,4]. Quasispecies dynamics is characterized by a continuous process of mutant generation, competition and selection, which results in the dominance of one or several genomes with high fitness surrounded by a mutant spectrum [5]. The population complexity of an RNA virus allows it to occupy a large adaptive landscape from which novel phenotypes may readily emerge, including rapid selection of mutant virions with decreased sensitivity to antiviral inhibitors. In the case of human immunodeficiency virus type 1 (HIV-1) different families of antiretroviral drugs have been used in clinical practice, including Nucleoside/Nucleotide Reverse Transcriptase Inhibitors (NRTIs), Non-Nucleoside Reverse Transcriptase Inhibitors (NNRTIs), Protease Inhibitors (PIs), Integrase Inhibitors (INIs), Fusion Inhibitors (FIs) and antagonists of CCR5 receptors. In the frame of HIV-1 quasispecies dynamics, non-suppressive treatment regimens trigger the selection of viral variants showing mutations and other genetic rearrangements that confer either resistance to individual drugs or multidrug-resistance [6,7].

As a result of the positive and negative interactions within mutant spectra that compose the evolving quasispecies, low-frequency viral subpopulations can be selected when environmental conditions change [8,9]. Additionally, experiments using foot-and-mouth disease virus (FMDV) in cell culture [10,11], and HIV-1 *in vivo* [12] showed that viral quasispecies may possess a molecular memory of their past evolutionary history maintained as minority components (ranging from 0.1% to 20% of the total number of genomes) within their mutant spectra. In HIV-1, quasispecies memory reflects the genomes that were dominant at an earlier phase of the intra-host evolutionary history. Such minority genomes are able to drive the ensuing evolution of the virus during chronic infections, particularly its response to antiretroviral treatments [13]. In HIV-1 infection, drug- and multidrug-resistant minority variants can also be stored in the form of proviral DNA [14,15]. It is still a matter of debate whether the presence of mutations that confer decreased sensitivity to antiviral drugs in minority HIV-1 genomes affects the *in vivo* efficiency of such inhibitors. The risk of treatment failure has been associated with the presence of low-frequency baseline resistant mutants in HIV-1 infected patients [16–24]. In turn, the impact of minority mutations within HIV-1 quasispecies has not been related with treatment failure in other cases [25–27]. However, from a virological point of view, the characterization of both majority and minority mutant genomes would be advisable in clinical practice to make HIV-1 genotyping a predictive tool regarding the expected viral sensitivity to antiviral drug combinations [3,28,29].

The traditional HIV-1 population or consensus sequencing provides information limited to the genotype of the predominant or major viral variant, and it fails to detect minority subpopulations represented in less than 25% of the total quasispecies [30,31]. In turn, the sequence analysis of a representative number of molecular clones (usually, 20 to 100) derived from the amplified viral population is a very labour-intensive method, poorly adapted to high-throughput analysis in clinical laboratories. Over the last decade, next-generation sequencing (NGS) and single-genome sequencing have revolutionized the genotypic analyses of viral quasispecies diversity, as they allow a deeper penetration (down to 0.5–1%) into the composition of the evolving mutant spectrum [32]. NGS has been successfully applied to the screening of drug-resistance mutations in HIV-1 minority genomes [31,33–35], provided that appropriate correcting algorithms have been implemented to exclude artefactual mutations introduced during the enzymatic amplification and analytical processes [36,37]. However, drawbacks of NGS-based techniques still limit their daily applicability in clinical laboratories. Such limitations include the long time required for completing a sequencing protocol, together with the need for skilled technical personnel and expert bioinformatics support (essential for handling very large sequence datasets, analysis and interpretation). Further, the lack of standard *in vitro* and *in silico* methods, as well as the high cost of the sequencing equipment restrains the use of NGS-based techniques in clinical practice [31,38].

In parallel, several alternative, allele-specific assays have been developed with enough sensitivity to identify low-level drug-resistant variants (usually representing 1–15% of the total population and, in some cases, as low as 0.01%). They include PCR and restriction enzyme cleavage, allele-specific real-time quantitative PCR, ‘quasispecies diving’ (based on a stepwise, specific amplification of minority variants), oligonucleotide ligation-based assays, PCR-restriction fragment length polymorphism, as well as different variants of the heteroduplex mobility and heteroduplex tracking assays (reviewed in [9,28,39–41]). However, the detection of one or a few mutations at a time limits the usefulness of these assays in clinical practice. In turn, microarray-based approaches allowed multiplexing allele-specific HIV-1 genotyping assays [42,43] and showed a good concordance with conventional genotyping methods [44]. The evolution of microarray technology includes primer extension methods based on arrays of immobilized HIV-1-specific oligonucleotides [45] and the development of simple but robust HIV-1 drug-resistance testing microarrays useful in rural African areas [46]. Nevertheless, conventional HIV-1 genotyping microarrays contain a limited and non-customizable number of oligonucleotide probes, and they do not inform about resistance-associated mutations present in low-abundance viral subpopulations.

Here we describe a novel genotyping DNA microarray platform that informs about a large variety of drug-resistance mutations and amino acid insertions present in majority and minority subpopulations of HIV-1 quasispecies. The developed microarray contains a panel of 160 optimized oligonucleotide probes complementary to viral genomic regions involved in resistance and multidrug-resistance to NRTIs, NNRTIs and PIs. A customized bioinformatics protocol has been implemented that includes a new normalization procedure and a stepwise filtering algorithm able to extract all the useful information contained in each hybridization experiment. Such a protocol has been developed using 17 clonal HIV-1 samples and selected mixtures of them at different ratios, and further tested with 57 HIV-1 clinical samples extracted from multi-treated infected patients. A high genotyping accuracy has been obtained, while minority subpopulations representing as low as 5–10% of the total viral quasispecies have been characterized in the analyzed clinical samples. Although some limitations of the developed microarray have been identified in its current version, its overall performance, ease of use and cost effectiveness makes this genotyping platform a useful tool for HIV-1 drug-resistance testing in daily clinical practice, thus paving the way for personalized antiretroviral therapies.

## Materials and Methods

### Origin of HIV-1 samples

Seventeen pure clonal HIV-1 samples were used as the genotyping microarray training set. Among them, 11 samples included the coding region of the full-length PR (amino acids [aa] 1–99) followed by the first 245 aa of the RT of the HIV-1 *pol* gene, as previously described [13,47,48]: 1.95c2, 1.95c4, 1.95c5, 1.95c9, 2.94c5, 2.94c24, 2.94c63, 2.94c64, 5.96c9, 6.95c8 and 9.95c10. Other clonal samples contained the RT sequence alone (aa 31–245) coding for different amino acid substitutions (together with additional silent mutations): pWT (wild type genotype corresponding to the HIV-1 HXB2 strain, GenBank accession number K03455), pINS (insertion T69SSS), L2.22 (A62V+T69SSS+K70R+V108I), D67N (A62V+D67N+T69SSS+K70R+V108I+Y181C), V75I (V75I) and V75T (V75T) [48–50]. The nucleotide sequences of the training set have been deposited in GenBank (accession numbers KT711098 to KT711114).

In turn, the test set contained 57 retrospective clinical HIV-1 subtype B samples from patients followed at the HIV Unit of Hospital 12 de Octubre (Madrid, Spain) between 1996 and 1999. All HIV-infected patients provided informed consent for the use of their blood and plasma samples in HIV research, as requested by the Spanish Law of Biomedical Research. Samples were stored at the blood plasma bank of the Microbiology Department of Hospital 12 de Octubre and they were fully anonymized before being processed for RNA extraction. Viral RNA was obtained from plasma samples of patients that experienced two or more treatment failures despite good adherence to treatment, as defined by viral load increase above 400 HIV-1 RNA copies/ml in at least two determinations. Their viral loads were distributed as follows: 5 samples showed >10^5^ HIV-1 RNA copies/ml; 20 samples, 10^4^−10^5^ copies/ml; 10 samples, 10^3^−10^4^ copies/ml; 4 samples, <10^3^ copies/ml; 18 samples, unknown viral load.

### Preparation of target DNAs for microarray analyses

HIV-1 DNA target molecules corresponding to the training set were amplified from the cloned sequences using Expand High Fidelity DNA polymerase (EHF, Roche), as specified by the manufacturer. PCR amplification of the full PR-coding region was carried out using the forward primer 5’PROT2HindIII-P (5’-TCAGAGCAGACCAGAGCCAACAGCCCCACC-3’, corresponding to nucleotides [nts] 2138 to 2167 of HIV-1 CAM-1 strain, GenBank accession number D10112) phosphorylated at its 5’-end, and the reverse primer 140RD (5’-CATTGTACTGATATCTAATCCCTGG-3’, complementary to positions 2969–2993). In turn, PCR amplifications of the queried codons of the RT-coding region involved the phosphorylated forward primers RT1-P (5’-CCAAAAGTTAAACAATGGCCATTG-3’, nts 2606–2629) or 55F-P (5’-CAAAAATTGGGCCTGAAAATCC-3’, nts 2694–2715), and the reverse primers 153RD (5’-TATTGCTGGTGATCCTTTCC-3’, nts 3009–3028), 13RD (5’-GTTCATAACCCATCCAAAGG-3’, nts 3232–3251) or 20RD (5’-ATTGACAGTCCAGCTGTCTTTTTCTGGC-3’, nts 3289–3316). Longer amplicons containing the PR-RT-coding regions were obtained by PCR amplification using as primers 5’PROT2HindIII-P and either 20RD or RT3333R (5’-CCACTAACTTCTGTATGTCATTG-3’, nts 3311–3333).

DNA targets corresponding to the test set were obtained from the 57 previously described HIV-1 clinical samples. Viral RNA was extracted from plasma samples using the ‘HIV Sample Preparation Module’ of the ‘ViroSeq HIV-1 Genotyping System’ (Applied Biosystems), as specified by the manufacturer. The extracted RNA was retrotranscribed and PCR amplified by means of the ‘OneStep RT-PCR Kit’ (Qiagen), since this system showed higher sensitivity for samples with low viral load than other two-step protocols essayed (data not shown). Primers used were 5’PROT1 (5’-AGGCTAATTTTTTAGGGAAAATCTGGCCTTCC-3’, nts 2080–2111) and 153RD for the PR region, and RT-INI (5’-ACAGTATTAGTAGGACCTACACC-3’, nts 2474–2496) and RT3333R for the RT region. The RT-PCR amplification of a single amplicon containing the PR-RT-coding regions involved primers 5’PROT1 and RT3333R, though this approach showed less efficiency for samples with viral load lower than 10^4^ copies/ml. Upon RT-PCR, nested PCR amplifications of the PR-coding region were performed with EHF, using the forward primer 5’PROT2HindIII-P and the reverse primer 140RD (positive amplification was successful in 53 out of the 57 samples), while those of the RT-coding region involved different combinations of the forward primers RT1-P or 55F-P, and the reverse primers 153RD, 13RD or 20RD (51 samples amplified). To ensure that bottlenecking effects were not present during the amplification of the circulating HIV-1 quasispecies it was checked that, in addition to reactions with undiluted DNA, nested-PCR amplification gave positive results from 1/10 and 1/100 diluted samples.

The consensus or population nucleotide sequence of the test set was determined using the Big Dye Terminator Cycle Sequencing Kit (Applied Biosystems) with ABI Prism 373 DNA Sequencer and 3730 DNA Analyzer (Applied Biosystems/HITACHI). The composition of the circulating quasispecies present in the clinical samples was determined by clonal analysis. To do so, the products of the nested PCR-amplified DNA were cloned using the TOPO TA cloning PCR II Kit (Invitrogen) and the recombinant plasmids were used to transform One Shot Mach1-T1 (Invitrogen) competent *E*. *coli* strain. The sequences of 13 to 34 molecular clones of both PR and RT regions of each clinical sample (total: 1,162 and 1,092 clonal sequences of the PR and RT regions, respectively) were obtained and analyzed (GenBank accession numbers KT711115-KT712275 and KT712276-KT713367).

### Microarray design and printing

Antisense DNA oligonucleotides complementary to HIV-1 sequences containing codons involved in drug-resistance and multidrug-resistance to PR and RT inhibitors were designed. They spanned the whole PR-coding region and the first 240 codons of the RT (positions 2270 to 3272 of the HIV-1 subtype B *pol* gene) (Table A in S1 File). Every queried codon was covered by at least one oligonucleotide containing the wild type sequence and at least one mutant oligonucleotide. Each oligonucleotide (whose name corresponded to the queried HIV-1 codon) included a ‘C6 amino linker’ group [NH_2_(CH_2_)_6_] at its 5’-end, a 15-mer spacer region with either (T)_15_ or (TCC)_5_ sequence and the specific, 13 to 17 nt-long sequence containing the queried codon at its central position. The (TCC)_5_ spacer sequence was used instead of the (T)_15_ one when the latter could induce partial self-complementarity with poly-A tracks present at the specific sequence. Sequence and length of the oligonucleotides were stepwise optimized in preliminary versions of the microarray (data not shown) to avoid formation of stable hairpins and to ensure a proper hybridization with the queried wild type (wt) and mutant codons in the context of their most common flanking regions, as shown in HIV-1 sequence databases (http://www.hiv.lanl.gov/content/sequence/HIV/mainpage.html and http://hivdb.stanford.edu). The calculated melting temperature of all the oligonucleotides was 49–55°C, most of them being in the range of 49–51°C (Table A in S1 File).

Four highly conserved HIV-1 sequences within the PR and RT-coding regions (spanning codons 25–29 and 94–99 of the PR, as well as 23–28 and 167–171 of the RT) were designed as internal hybridization positive controls (IHC) (Table A in S1 File). Two unrelated oligonucleotides, lacking any sequence homology with the target HIV-1 region, where included in the study as negative controls. They are termed G142-15r (whose sequence is complementary to codons 3625 to 3639 of foot-and-mouth disease virus VP1 coding region) and E142-15r (corresponding to the same FMDV region, with the mutation C3632T). Both control oligonucleotides had previously shown a good performance in DNA microarrays [51]. Thus, a total of 160 different probes were synthesized and HPLC-purified (Sigma): 154 probes specific to the queried HIV-1 codons, 4 positive controls and 2 negative controls. Among the specific probes, 37 of them corresponded to different variants of 11 PR codons, while 117 covered variants of 31 RT codons including insertions and deletions in the region 67–70 as well as the multidrug complex associated to the mutation Q151M [6].

All oligonucleotides were diluted in 1× spotting solution (Telechem-Arrayit) at 50 μM final concentration, and spotted onto super-epoxy-coated glass slides (Telechem-Arrayit). Spots containing spotting solution with no oligonucleotide were used as additional negative controls. Microarrays were printed using a GMS 417 Arrayer (Genetic Microsystems) and defined four grids or subarrays per slide. Each subarray contained 360 spots arranged in two regions (PR and RT), every oligonucleotide being printed in duplicate spots of 150 μm in diameter, with a center-to-center distance of 250 μm (Figure A in S1 File). Each hybridization assay involved two adjacent subarrays, thus producing four replicates in every probe-target hybridization. Thus, each printed microarray allowed two independent hybridization experiments.

### Microarray hybridization and scanning

Before hybridization, printed microarrays were washed in 2× saline-sodium citrate (SSC) buffer and 0.1% N-lauryl sarcosine, for 2 min at room temperature, followed by 2 additional min in 2× SSC, as described [51,52]. This step allowed removing unbound DNA from the microarray surface and washing the excess of spotting solution. Printed oligonucleotides were denatured by baking the slides for 2 min in boiling milli-Q water and cooling for 10 sec at room temperature. The oligonucleotides were immediately fixed by plunging the slides into ice-cold 100% ethanol for 2 min, followed by centrifugation at 500×g for 1 min (using an Arrayit minicentrifuge). Microarrays were prehybridized with 20 μl of hybridization buffer (6× SSC, 0.5% SDS, 1% BSA) for 45 min at 42°C, under a 24 × 24 mm cover slip in a hybridization chamber (Genetix). Finally, the microarrays were washed with distilled water, and dried by centrifugation.

The phosphorylated strand of the HIV-1 DNA targets to be hybridized was specifically degraded using lambda exonuclease (New England Biolabs), and the resulting ssDNA was fluorescently labeled with Alexa Fluor 647, using the Ulysis Alexa Fluor 647 Nucleic Acid Labeling Kit (Life Technologies) as described [51,53]. Hybridization with the labeled target (50 ng, equivalent to 0.3 pmol Alexa Fluor 647) was carried out in hybridization buffer at 50°C for 3 h, using microarray hybridization chambers (Genetix). Hybridized microarrays were washed at 45°C in 2× SSC and 0.1% N-lauryl sarcosine for 5 min, then in 2× SSC for 5 min, rinsed in 0.2× SSC for 10 sec, and finally in distilled water for 5 min. Slides were dried by centrifugation at 500×g for 1 min and immediately scanned at 635 nm using either GenePix 4000B (Molecular Devices) or G2565AA (Agilent) high resolution microarray scanners. The reproducibility of the method was assessed in preliminary experiments (not shown) by comparing the results of at least five different hybridizations for both PR and RT regions. Microarrays were excluded from further analysis when yielding a high background, displaying uneven fluorescent signal, lacking hybridization signal in the corresponding (PR or RT) HIV-1 positive control spots, or showing hybridization signals in any of the FMDV negative controls.

### Bioinformatics data processing

#### Datasets

The training set included microarray hybridization data from 17 pure HIV-1 clonal samples (268 hybridization experiments) as well as mixtures of two of these clonal samples (either pWT/pINS or 1.95c9/2.94c64) at different ratios (in %): 0/100, 1/99, 5/95, 10/90, 50/50, 90/10, 95/5, 99/1 and 100/0 (110 hybridizations). The test set was composed of hybridization data of HIV-1 quasispecies successfully amplified from 53 (PR) and 51 (RT) clinical samples (119 hybridization experiments).

#### Image analysis and quantification

Hybridization images were processed using GenePix Pro 6.0.0.68 (Molecular Devices) software in GenePix Scanner, and ScanArray Express 2.0 (Perkin Elmer) in Agilent one. No automatic normalization of images was performed. A number of variables corresponding to the scanned spots were quantified by both software packages, including the median fluorescence intensity in each spot and its respective local background. Additionally, the microarrays scanned using the GenePix system contained information about the number of background pixels (BP), while microarrays processed using the ScanArray platform showed information about the diameter of the spot (DS) and the percentage of foreground signal higher than the background minus its standard deviation (BSD).

#### Spots quality control

A stepwise quality control protocol was implemented to exclude low quality hybridization signals from further analyses. Quality was evaluated at four different stages: individual spots, overlapping probe signals, duplicated probes and full microarrays. Initially, spot filters based on either BP or DS were implemented to remove the experimental noise arising from hybridization experiments and not due to biological variation. The election of such filters was based on a preliminary study of the distribution of quantified hybridization data binned as correctly classified or not (with respect to the theoretical hybridization tables, see below). Spots which did not satisfy this quality control were not considered for further analysis. The remaining quality control stages are explained below.

#### Normalization of hybridization signals

Normalization of the data has been recognized as a necessary pre-processing step in a variety of high-throughput technologies, including DNA microarrays. All known normalization methods rely on assumptions about data features that are expected to be invariant across samples. Different normalization protocols have been developed for gene expression microarrays [54], though there is no consensus on their relative performance on genotyping microarrays containing a relatively small number of oligonucleotide probes. However, the incorporation of an accurate normalization method in the analysis was required to trace the array-to-array variability in this study. The selected raw fluorescence value for each spot was the median foreground signal after subtracting its local background. Technical replicates for each target-probe hybridization (4 spots per experiment) were clustered together. It was assumed that some of the probes spotted onto each microarray specifically hybridized to their fully complementary sequences present in the (either unique or majority/minority subpopulation of) labelled target DNA, thus rendering a positive signal. In the event that any other residual hybridization signal was present, it was called negative signal or noise. Therefore, normalization was carried out using a stepwise method. First, since two different classes of signals (positive or negative) are expected, they were clustered into two groups using the K-means algorithm [55–57]. Then, after removing the outliers from the positive group by applying the Grubb’s test [58–60] with a significance level of 0.05, the mean of the positive signals was obtained. Since most of the hybridization experiments have been performed using target DNA amplicons including either the PR or the RT-coding sequences of the *pol* gene, two regions (containing the PR and RT probes, respectively) were separately normalized in each microarray by means of their own mean positive signal. These two values were used as the normalization factor for each probe by applying the following function:

$$ǁPSǁ=\sqrt{\frac{\sum_{i=1}^{N^{RP}}I_{i}^{RP}/N^{RP}}{\sum_{i=1}^{N^{PS}}I_{i}^{PS}/N^{PS}}}$$

where ǁ*PS*ǁ is the normalized probe signal, *N*^*RP*^ is the total number of replicas per hybridized probe (typically, 4), *I*^*RP*^ is the signal intensity of a replica probe *i*, *N*^*PS*^ is the total number of positive probe signals per array region (either PR or RT), and *I*^*PS*^ is the intensity of a positive probe signal *i* within such a region.

#### Theoretical hybridization tables

To evaluate the classification accuracy of the method, tables showing the theoretical hybridization signal of each probe with each hybridized target molecule (the previously sequenced samples of the training set) were required. The theoretical hybridization tables were based on the sequence complementarity between probes and targets: a positive hybridization signal was expected when probe and target are fully complementary, while the presence of any single mismatch between them (including those at the 5’ or 3’ ends of their hybridizing sequences) was assigned to a negative signal. Therefore, partial hybridizations were not allowed in the theoretical tables, assuming that this stringent criterion would assign any incomplete hybridization events that resist the washing step (e.g., those including one or two mismatches between probe and target) to false positive signals. In turn, when the hybridized target sample contained a binary mixture of two HIV-1 clones from the training set, a theoretical consensus table was generated based on the minimum percentage of each sequence in the mixture that could be detected. This allowed defining a preliminary threshold value for each probe, which was further used for the genotyping of clinical samples belonging to the test set.

#### Probes calibration

Although the 160 spotted probes were designed to show similar theoretical hybridization temperatures (Table A in S1 File), not all of them behaved equally in the high throughput hybridization experiments performed. Thus, it was necessary to characterize the response profile of each probe when it hybridized with either its specific target sequence or an unspecific one. To do so, the normalized hybridization data produced by the training set was reorganized into groups of positive or negative hybridization signals for each probe, based on the theoretical hybridization tables previously built. The density of normalized intensities showed that both signal and noise data could be modelled by distribution functions for each probe. Particularly, normal and log-normal distributions were fitted to positive and negative normalized datasets, respectively. Such distributions are expected to be centred at 1 for positive signals and at 0 for negative signals. Distribution functions parameters were used in further steps to assess the probability of any observed signal to belong to a positive or negative hybridization event. For some probes, the characterization of one type of signal (either positive or negative) was missing due to the absence of this information in the training set (e.g., a probe containing a mutant codon not hybridizing to any of the available target molecules). In such cases, the average information derived from the whole training set was used: all the available normalized data was divided into 2 groups (positive and negative signals) based on the theoretical hybridization tables, and their densities were adjusted to normal and log-normal distributions (termed general reference curves).

#### Probes quality control

The poorly discriminant probes were discarded based on the overlapping area between positive and negative distribution functions, quantified for each probe by integral calculation. Probes whose overlap was higher than 25% were not considered for further analysis. Additionally, during the development of consecutive versions of the HIV-1 genotyping microarray, different probes complementary to certain nucleotide regions of the viral genome (e.g., those including a given resistance-associated codon in the context of alternative flanking sequences) were designed and tested. Because of their good performance, some of the probes containing the same interrogating codon were maintained in the final version of the microarray. Thus, to avoid redundant information we selected, in each hybridization experiment, the equivalent probe that showed the lowest overlap between its positive and negative distribution functions.

#### Full microarrays quality control

Once all useful information from the hybridized microarrays had contributed to the quality control of the probes, the last step consisted in excluding those microarrays showing an excess of positive signals. The spotted probes explored a variable number of codons for each position of interest in the PR or RT regions of the *pol* gene: on average, a probe for each wt codon sequence and two probes for its alternative mutants. Since the expected number of positive signals for any sample of the training set should be around 1/3 of the probes included in each microarray, those showing a higher number of hybridization signals were discarded.

#### Classification and evaluation of clinical HIV-1 samples

Once a clinical sample from the test set was hybridized to the microarray, the cumulative probability difference to belong to either the positive or negative distribution was computed for each normalized probe signal. Probabilities were based on the distribution functions previously obtained for each probe using the training set. As stated above, when either positive or negative hybridization data were lacking for a given probe, the general reference curves were used. When the absolute cumulative probability difference was smaller than 0.05, the signal was classified as ‘undefined’ because of the high chance of a wrong classification. A theoretical hybridization table was built for clinical HIV-1 samples of the test set, based on the previous sequencing and alignment of a number of clones (13 to 34) of each sample, assuming the preliminary detection threshold per probe derived from the hybridization of binary mixtures (training set). Thus, to evaluate the classification accuracy of the method for clinical samples, once a signal was classified as positive or negative, it was checked if that was in agreement with the theoretical hybridization table. This rendered the final classification of each hybridization signal as true positive (TP), true negative (TN), false positive (FP), false negative (FN) or undefined (UD), and allowed defining the accuracy and sensitivity of the developed genotyping microarray for detecting minority subpopulations within HIV-1 quasispecies in clinical samples.

## Results

### Optimization of the genotyping microarray

Preliminary experiments based on our previous experience [51,61–63] were conducted to compare the performance of control microarrays that were hybridized using different experimental parameters (data not shown). Regarding the preparation of the target molecules, control experiments advised against the use of streptavidin-coated magnetic beads to obtain ssDNA by discarding the biotin-labelled strain. Also, our results showed a lower labelling efficiency when either Cy3-dUTP or Cy5-dUTP were incorporated during DNA amplification. With respect to the spotted probes, a lower overall performance was observed when the (T)_15_ and (CCT)_5_ spacers were either not included or shortened (range tested: 7–13 nts), when the interrogating region of the oligonucleotide was shorter than 15 nts (range tested: 9–14 nts), and when the queried codon occupied positions outside the interval 7–11 within the interrogating 15-mer. The nucleotide sequence of the spotted probes was also stepwise optimized based on their individual performance in five successive versions (termed I to V) of the microarray: for each codon, only the probes showing the highest performance were maintained in the final version of the genotyping microarray (Figure A in S1 File**)**. Additionally, preliminary experiments showed a decrease of the overall hybridization efficiency when aldehyde-coated slides were used, probe oligonucleotides were spotted at concentrations lower than 50 μM (range tested: 5–45 μM), hybridization solution contained 50% formamide, and hybridization time was shorter or longer than 3 h (range tested: 1 to 4 h). Microarray hybridization and washing temperatures were also optimized for point mutation discrimination in systematic preliminary experiments (ranges tested: 45–58°C and 40–55°C for hybridization and washing steps, respectively). As a result, the use of the optimized amplification, labelling and hybridization conditions described in Materials and Methods rendered useful hybridization data for most of the clinical samples (53 and 51 successful amplifications out of the 57 initial samples for the PR and RT, respectively) belonging to the test set, irrespectively of their viral load. Indeed, the discarded clinical samples did not produce amplification by RT-nested PCR in any of the conditions and primer combinations assayed, probably due to the presence of a high number of mutations in their primer-binding regions. The whole set of raw microarray hybridization data used in this study has been uploaded to Gene Expression Omnibus (GEO) repository (https://www.ncbi.nlm.nih.gov/geo/), with the accession number GSE90621.

Two examples of microarray hybridizations of wild type PR and RT samples using the optimized experimental conditions described in Materials and Methods are shown in Figure A in S1 File. All the wild type probes showed a clear hybridization signal in these examples, except in cases where the hybridized sample contained a variant genotype in a given region, not fully complementary to the spotted probe (positions M36-PR in panel B as well as L210-3 and M230-3 in panel C). Also, probes corresponding to RT codons 41 (panels B and C), 236 (B) and 238 (B) could not hybridize because the labelled target molecules did not include them. No cross hybridization with the respective mutant probes was observed (except in positions N67-2 and N70a of panel C), signals were produced in the corresponding positive control probes (IHC-PR3 and IHC-RT4) and no hybridization signals appeared in any of the negative controls.

Other examples of hybridized microarrays included signals produced by different mutant targets belonging to the training and test sets. Figure B in S1 File shows hybridization signals at different mutant PR probes (I36, I46, L46, V48, V54, V71, T82, V84, M90 and M90-2) and at mutant or insert-containing RT probes (V62, S68, S69, Ins69a, Ins69b, Ins69c, Ins69d, I75, T75, I100, E101, I108, M151, M178, C181, I184, H188, K211, Y215, C215 and T238). In turn, examples of microarrays hybridized with targets belonging to the test set that contained majority and minority variants are shown in Figure C in S1 File. These examples have been chosen to exemplify concordant and discrepant results among the detected hybridization signals, the percentages of variants derived from clonal analysis, and the amino acid sequence present at the queried codon as predicted by population sequence of the sample.

### Quantification of microarray hybridization signals and quality control

To quantitatively analyse the performance of the genotyping microarray in terms of accuracy, specificity and sensitivity, a detailed bioinformatics analysis was designed and carried out. It was assumed that, although a high number of interrogating probes were tested and optimized along the different versions of the microarray, some probe-target hybridizations could be either hindered or impeded due to nucleotide mutations eventually present in the sequences of the training set at positions adjacent to the queried codons. Thus, once the consensus sequences of the samples were obtained, the sequence complementarity between the spotted probes and the hybridized targets was evaluated. This allowed constructing the theoretical hybridization tables of the training set (Figure D in S1 File).

Then, a stepwise filtering protocol was applied to the hybridized microarray signals from samples belonging to the training and test sets. The first filtering criterion involved individual spots quality. The appropriate cut-offs were selected based on control experiments (not shown) that compared the hybridization with targets from the training set with the expected results derived from the theoretical hybridization tables. As a result, microarray spots scanned with the GenePix platform passed the filter if BP > 300, a criterion that discarded most of the FP but also some of the TP signals. In turn, spots of microarrays analysed with the ScanArray platform were accepted if either DS ≤ 150 μm, or DS > 150 μm being BSD ≤ 40: these criteria eliminated FP signals while avoiding the filtering of TN ones (Figure E in S1 File). Using these filters, 0.20% of all hybridized spots from the training set were discarded, while 1.27% (RT region) and 4.05% (PR region) of spots were discarded from the test set (Table 1).

**Table 1:** Summary of the stepwise filtering protocol applied to the hybridized microarrays.

Quality control step	Feature	Discarded features (%)	
		*Training set*	Test set
**1: Spot filter**	Individual spots	*0*.*20*	4.05 (PR spots); 1.27 (RT spots)
**2: Probe filter 1**	Probes overlapping >25% *	*9*.*09*	9.09
**3: Probe filter 2**	Duplicated probes *	*10*.*13*	10.13
**4: Array filter**	Full microarrays	*4*.*46*	4.16 (PR region); 0 (RT region)

* The discarded probes in the training set were not used in the test set, while no extra probes were discarded in the test set. As a result, the % of discarded probes is equal in both sets.

**Fig 1:**
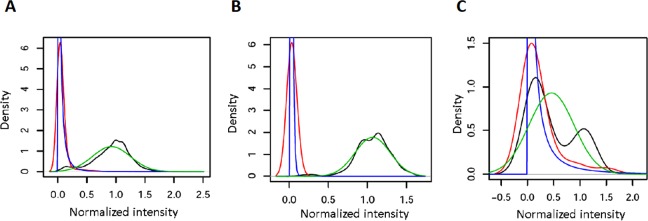
Examples of the density of normalized hybridization signals from the training set and their corresponding distribution functions for positive and negative data. A) General reference curves; B) Probe-specific curves for probe Y188a, showing no overlap between positive and negative distribution functions. C) Probe-specifc curves for probe M230-3, with high overlap between positive and negative distribution functions (probe discarded during the quality control, see text). The probe-specific curves for the 124 probes that passed the quality control are shown in Figure G in S1 File. Color code: Red, density of normalized negative hybridization signals; Blue, fit of the negative data to a log-normal distribution; Black, density of normalized positive hybridization signals; Green, fit of the positive data to a normal distribution.

The fluorescence signal of every probe was normalized with respect to the mean positive signal of each array region (PR or RT). Such normalized probe signals ǁ*PS*ǁ allowed discriminating between positive and negative hybridizations in most cases (Figure F in S1 File). Then, positive and negative normalized hybridization datasets for every probe were independently fitted to normal and log-normal distributions (probe-specific curves), respectively (Figure G in S1 File). In parallel, general reference curves were constructed for circumventing the lack of positive and negative signal calibrations for some of the probes included in the microarray. Indeed, the hybridization of the training set showed that 52 probes (32.90%) produced either positive or negative signals in different experiments, 9 probes (5.70%, mainly wt) rendered only positive signals, and 97 probes (61.40%, mainly mutants) produced only negative signals. The general reference curves, together with two examples of probe-specific curves showing high and low discrimination power, are shown in Fig 1. Thus, the combination of positive or negative probe-specific curves with negative or positive general reference curves, when required, further allowed the classification of signals not previously characterized in the training set. The combination of such general and probe-specific distribution functions was possible because their parameters were similar: the mean of positive distributions was 0.92 and 0.91 (with standard deviations of 0.33 and 0.30) for general and probe-specific fits, respectively, while the mean of negative distributions was -3.32 and -3.24 (mean standard deviations of 1.13 and 0.99), respectively.

Positive and negative distribution functions presented certain degree of overlapping in some of the probes. Thus, though 76% of probes discriminated correctly (overlapping <10% between the positive and negative distribution curves), 24% of probes showed curves overlapping >10%. Such distinct behaviours were exemplified by probes Y188a and M230-3 in Fig 1B and 1C. When a maximum overlap of 25% between fitted curves was allowed, 14 probes (9.09% of the total discriminant ones, being M230-3 one of them) were discarded. Moreover, when two probes had been designed for detecting the same codon in different sequence backgrounds, only the one with the lowest overlap between positive and negative curves was maintained. Following this criterion, 16 additional probes (10.39% of the total) were excluded (Table 1). Therefore, as a result of the two-steps probes quality control, the number of discriminant probes remaining for further analysis (of both the training and the test sets) was reduced from 154 (37 of them belonging to the PR region and 117 to the RT) to 124 (29 in PR and 95 in RT, Figure G and Table A in S1 File).

The information derived from the spots that passed the first filtering step was used to filter the probes showing a suboptimal performance. Then, the last quality control step allowed discarding the whole hybridized microarrays when an excess of positive signal was produced due to unspecific hybridization. To do so, both the number of positive signals per microarray and the related false positive vs. false negative ratios were plotted (data not shown). This allowed establishing that microarrays with more than 12 and 35 positive signals at the PR and RT regions, respectively (around 1/3 of the spotted probes), had to be excluded from further analysis. Thus, 4.46% of the microarrays that were hybridized with the training set and 4.16% of those hybridized with the test set in the PR region were discarded, while all the microarrays hybridized with the test and training sets in the RT region passed this last filtering step (Table 1).

### Accuracy in viral genotyping

The overview of the classification of all hybridization signals before filtering is shown in Table 2. The main source of errors in the training set, the PR and the RT test sets (5.09%, and 9.06% to 9.80%, respectively) were FP signals appearing at probe positions not expected to show any signal as the sequences of probe and target are not fully complementary (see Figure D in S1 File). In turn, FN signals were scarce in the training set (1.33%), whereas their value increased to 2.72% (RT region) and 5.41% (PR) in the test set. Finally, UD spots were found at levels below 2.15% in both sets.

**Table 2:** Genotyping accuracy of the microarray with samples from the training and the test sets, calculated by comparing the experimental hybridization signals with the expected signals derived from the theoretical hybridization tables.

Signals	Classification accuracy (% of signals) before filtering		
	*Training Set(PR+RT)*	Test Set	
		PR	RT
**Correct**	***93*.*04***	**84.42**	**85.27**
**False Positives (FP)**	*5*.*09*	9.06	9.80
**False Negatives (FN)**	*1*.*33*	5.41	2.72
**Undefined (UD)**	*0*.*54*	1.11	2.15

The increased classification performance upon the 4-step, sequential filtering protocol applied is shown in Table 3. For the training set, the percentage of correctly classified signals (i.e. accuracy) increased up to 96.33% upon filtering, while for the test set the accuracy reached 93.53% and 89.24% in PR and RT regions, respectively. Consequently, the filtering protocol was more effective for the PR signals than for the RT signals of the test set. Among the different kinds of errors, both FP and FN signals showed a neat reduction during the stepwise filtering, while UD signals were less affected by the process. The classification accuracy in the training set after the filtering protocol is graphically depicted in Figure H in S1 File, where probes that still accumulated most of the FP signals (*i*.*e*. I46-PR, K65-2, D67a-2 and E219) and FN ones (Y181) were clearly identified.

**Table 3:** Genotyping accuracy of the microarray after the stepwise filtering approach.

Signals	Classification accuracy (% of signals)											
	Step 1: Spot filter			Step 2: Probe filter 1 (overlapped)			Step 3: Probe filter 2 (duplications)			Step 4: Array filter		
	*Trai-ning set*	Test set		*Trai-ning set*	Test set		*Trai-ning set*	Test set		*Trai-ning set*	Test set	
		PR	RT		PR	RT		PR	RT		PR	RT
**Correct**	***94*.*01***	**86.00**	**86.09**	***96*.*37***	**92.42**	**88.48**	***95*.*43***	**92.14**	**89.24**	***96*.*33***	**93.53**	**89.24**
**FP**	*3*.*99*	7.62	9.54	*2*.*63*	4.70	7.14	*3*.*06*	4.89	6.87	*2*.*19*	3.84	6.87
**FN**	*1*.*61*	5.39	2.50	*0*.*52*	2.21	2.40	*1*.*02*	2.13	2.26	*1*.*07*	2.16	2.26
**UD**	*0*.*40*	0.99	1.87	*0*.*48*	0.67	1.99	*0*.*49*	0.85	1.64	*0*.*41*	0.48	1.64

The distribution of genotyping errors per probe was further analysed in all the hybridised clinical samples belonging to the test set (Figure I in S1 File). It is evident that most of the errors were accumulated in a limited number of probes with partially overlapping curves (in the range 10–25%) that were not excluded during the filtering process, as exemplified by probe R103-3 (overlapping area of 11.3% and 18 classification errors). However, in probes Y188a and V108-2 the source of errors (18 and 20, respectively) was independent of the overlapping area between their fitted curves. Of interest, none of these three probes accumulating the highest number of errors is especially relevant for genotyping HIV-1 drug-resistance variants. In turn, a subset of the hybridized clinical samples was responsible for most of the errors (in particular A23, A26, A44 and A55, with 22 errors each of them, Figure I in S1 File), due to the fact that the HIV-1 quasispecies circulating in those multi-treated patients accumulated a high number of mutations at positions adjacent to the queried codons.

### Sensitivity of detection for minority HIV-1 subpopulations

**Fig 2:**
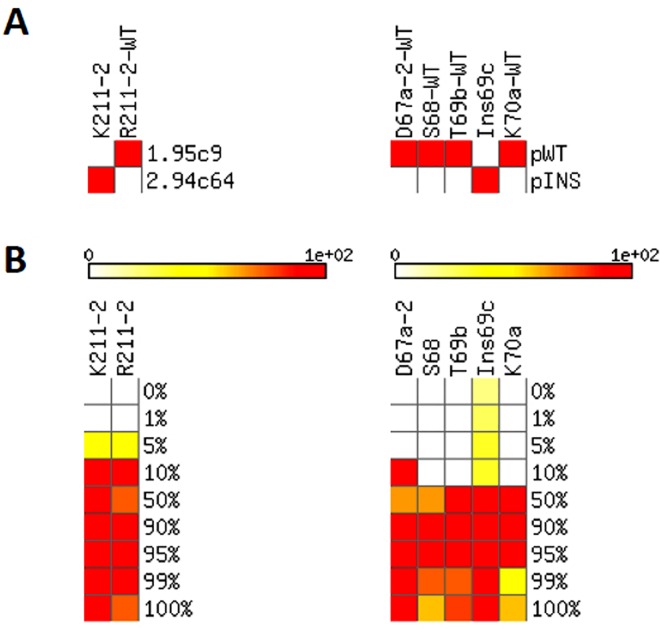
Detection sensitivity, estimated based on binary mixtures of pure samples. A) Theoretical hybridization tables of the pure samples used in the mixtures (1.95c9/2.94c64 and pWT/pINS) presenting 7 discriminating probes. Color code: Red, expected positive hybridization; White, expected negative hybridization. B) Rate at which each sample in the mixture produces a positive hybridization with each probe identified in panel A. Bar shows the transition from white (positive signal not detected) to red (positive signal detected in all the hybridization experiments).

Binary mixtures of pure clonal samples at known ratios were used for a preliminary exploration of the sensitivity of this microarray at detecting minority subpopulations in HIV-1 quasispecies. These mixtures involved either samples 1.95c9/2.94c64 (62 microarrays) or pWT/pINS (48 microarrays). The theoretical hybridization tables of these pure samples and their mixtures are shown in Figure J in S1 File, and the 7 non-redundant probes at which a differential hybridization is expected at the RT region between the samples in each mixture is depicted in Fig 2A. The hybridization of these binary mixtures with the microarray, after the corresponding stepwise filtering process of the signals (Table B in S1 File), produced the results summarized in Fig 2B.

Each of the probes presented a characteristic detection sensitivity for minority genomes. In the mixture 1.95c9/2.94c64, probes K211-2 and R211-2 showed positive signals in all hybridization experiments where their specific targets were present at percentages of at least 10%. Furthermore, in 50% of the experiments (31 of 62 microarrays) these probes showed a neat hybridization signal when their specific targets were present at proportions of 5% in the mixture. In turn, using the mixture pWT/pINS, 4 out of the 5 selected discriminating probes showed a positive signal when their specific targets were present at percentages ranging from 10% to 50%, being D67a-2 the only probe able to detect minority genomes at proportions of 10%. Unfortunately, the probe Ins69c produced FP signals even when its specific target (pINS) was not present in the mixture (as anticipated by the overlapping between their positive and negative distribution functions, Figure G in S1 File).

Given the variable detection thresholds obtained for the different probes analysed, as well as the limited number of available mixtures of pure clonal samples, a cut-off of 10% was set as the preliminary sensitivity threshold for the detection of minority subpopulations within the quasispecies. This value was used to build the theoretical hybridization tables of the clinical samples belonging to the test set once their quasispecies composition was analysed by clonal sequencing (see below). Still, the results obtained with the mixture 1.95c9/2.94c64 suggest that the maximum sensitivity of the microarray for detecting minority genomes could reach a value of 5%.

### Detection of minority variants in clinical HIV-1 samples

Before hybridizing the microarrays with the test set samples, the genetic variants present in the quasispecies at each queried codon were characterized by sequencing between 13 to 34 clones (mean: 22) isolated from each sample. After aligning the sequences, the percentage of clones containing each queried codon was quantified, and the results were graphically depicted for the PR and RT regions (Figure K in S1 File). Among the 970 events of perfect probe-target matching in the whole test set, 92.5% of them corresponded to signals produced by majority sequences accounting for 90 to 100% of the quasispecies (Figure K in S1 File). Remarkably, 7.5% of the probe-target complementarities were due to minority sequences present at proportions lower than 20% in their corresponding sample. The distribution of these minority subpopulations in three intervals (1.00–4.99%, 5.00–9.99% and 10.00–19.99% of the quasispecies) is shown in Table C in S1 File. The theoretical hybridization tables corresponding to these clinical samples were constructed, assuming a sensitivity detection threshold preliminarily set to 10% (Figure L in S1 File).

**Fig 3:**
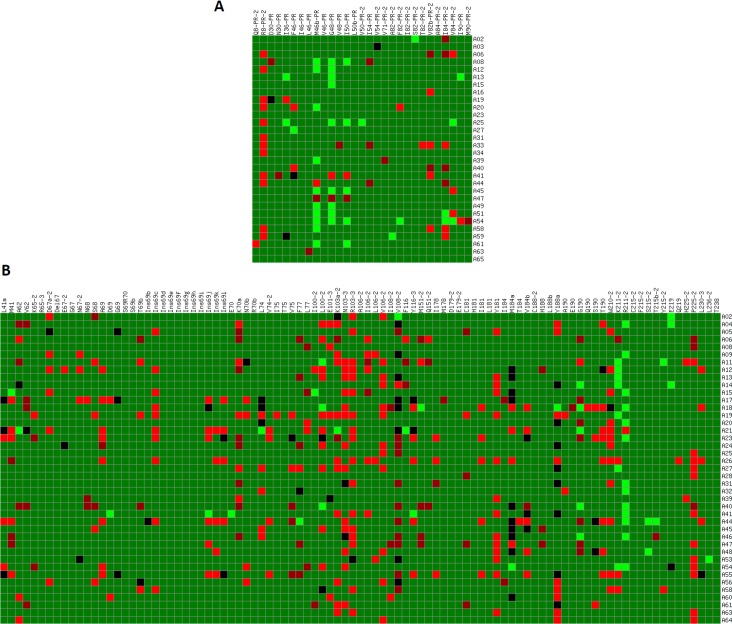
Classification accuracy of the hybridized clinical samples that contained minority subpopulations within their mutant spectra. Columns: probes included in the microarray corresponding to the PR (A) and RT (B) regions that passed the stepwise filtering protocol. Rows: hybridized samples. Color code: Dark green, correctly classified signal (TP or TN); Dark red, FN signal; Red, FP signal; Black, UD signal; Light green, no data (i.e. individual spots discarded during the quality control or by probe absence in some versions of the microarray).

**Fig 4:**
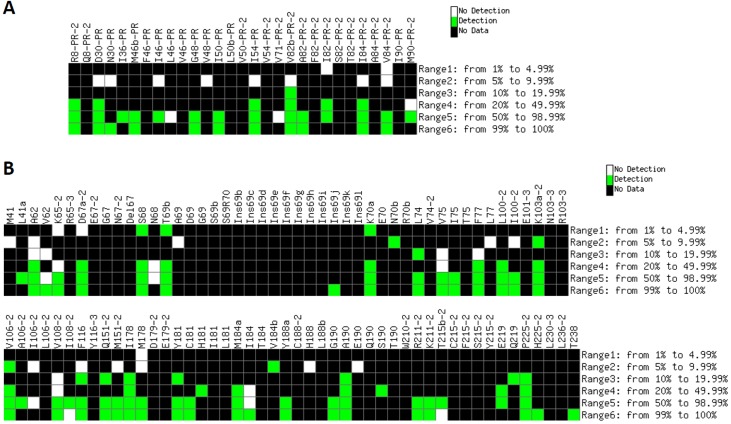
Detection of minority variants in clinical samples by the genotyping microarray. Columns: probes included in the microarray belonging to the PR (A) and RT (B) regions that passed the stepwise filtering protocol. Color code: Green, hybridization signal produced at the corresponding probe when the complementary target is present within a given rate (shown on the right side of each panel) in the quasispecies; White, lack of detection of an expected signal; Black, no data available (the sequence complementary to the queried codon is not present in any of the clinical samples at the corresponding percentage range).

All clinical samples (test set) were hybridized to microarrays, and the resulting signals were filtered using the previously described stepwise protocol. Then, the comparison of the theoretical hybridization tables (Figure L in S1 File) with the experimental hybridization data showed the classification accuracy of the microarray (Fig 3). Overall, 72.1% of all the expected positive signals in PR probes, and 79.5% in RT ones were successfully detected by the developed microarray (Fig 4). In the PR region, minority subpopulations in the interval 1.00–9.99% could not be detected, while those represented at proportions of 10–49.99% where correctly detected in 85.7% of the cases. In turn, probes corresponding to the RT region showed higher sensitivity for detecting minority subpopulations at proportions of 1.00–4.99% (41.2% of the cases), 5.00–9.99% (53.8%), and 10.00–49.99% (79.2%). Additionally, only 6 of the probes failed in the detection of variants present at proportions ≥ 50% (L46-PR, V71-PR-2, V62, N68, I106-2 and I184), and 2 FN signals were produced in probes I108-2 and T215b-2 when hybridizing with targets that contained their complementary sequences at proportion ≥ 99%. Noticeably, no FP signals were detected in any of the hybridization experiments involving clinical samples.

Regarding the performance of individual probes, it was evidenced that even those probes showing a limited sensitivity to detect minority genomes in binary mixtures of pure clones (S68, T69b and K70a, Fig 2B) did hybridize with clinical samples that contained the complementary sequences at proportions of only 1.00–4.99% of the quasispecies (e.g., sample A20, Fig 3B). Therefore, although the use of binary mixtures of clonal samples was useful to set a preliminary detection threshold to 10%, such value did not define the maximum sensitivity of the microarray for detecting minority subpopulations in clinical HIV-1 samples.

## Discussion

High-throughput, cost-effective, accurate and sensitive HIV-1 drug resistance detection assays are currently needed in the clinical practice. Although the detection of resistance mutations in minority HIV-1 subpopulations has not been unequivocally associated to a better prognostic value and a more efficient design of treatment strategies [25–27], a growing number of studies have linked the risk of treatment failure with the presence of low-frequency drug-resistant mutants in the circulating HIV-1 quasispecies [16–24]. Therefore, novel genotyping platforms should be able to detect and quantify the presence of drug- and multidrug-resistance mutations both in majority and minority genomes within the intrahost population. Over the past decade, two types of HIV-1 genotypic assays, sequencing-based and allele-specific, have been developed to detect point mutations related to antiviral drug-resistance. Conventional sequencing-based methods, including commercially available HIV-1 genotyping assays TruGene [64] and ViroSeq [65], have been widely used in the clinical practice. They detect all the mutations present in the analyzed consensus sequence of the viral population, though they are not sensitive enough to detect low-abundance mutations below a threshold of 25–30% [30,31]. Currently, NGS-based approaches are highly sensitive for detecting drug-resistance mutations in minority subpopulations [32,34,35], though these assays require highly technical/bioinformatics skills and are still too expensive to be a realistic option in most clinical laboratories. Indeed, the conventional genotyping and NGS-based commercial tests used for the detection of HIV-1 resistant variants have been recently discontinued. Therefore, alternative genotyping technologies are being requested by many different clinical HIV-1 departments that have no access to home-made genotyping tests.

In turn, DNA microarray technology has been explored as a fast, cheap and straightforward HIV-1 genotyping approach that, in combination with a customized bioinformatics analysis, can be adapted to high-throughput detection of low-abundance mutations in clinical practice. In the present study, we have developed a genotyping microarray that contains 160 oligonucleotide probes (optimized along different versions of the microarray) complementary to HIV-1 codons involved in drug- and multidrug-resistance to PIs, NRTIs and NNRTIs. Of interest, not only nucleotide mutations but also codon insertions and deletions have been included in the spotted oligonucleotide variants. 17 clonal HIV-1 samples and selected mixtures of them at different ratios have been used as the training set, while 57 HIV-1 clinical samples extracted from multi-treated infected patients constituted the test set.

Once the hybridization protocol was optimized, the developed bioinformatics filtering protocol allowed discarding a number of low quality individual spots and probes without compromising the number of good quality, full microarrays to be kept for analysis. Therefore, such a stepwise procedure maximized the recovery of useful information from the experimental data. Indeed, before applying such filtering, 9.7% of the microarrays that were hybridized with samples from the training set and 12.6% of those hybridized with clinical samples (8.4% of the PR region and 4.2% of the RT) showed an excess of positive signals that would have recommend their elimination. Nevertheless, after the individual filtering of spots and probes, the amount of full microarrays to be discarded decreased to 4.46% in the training set and 4.16% (PR arrays) or even 0% (RT arrays) in the test set (Table 1).

It became evident that quality control filters must be applied sequentially and following a specific order to maximize classification accuracy as each filter progressively and differentially decreases the rate of FP and FN signals. Thus, with our proposed four-steps sequential filtering protocol, the ratio of correctly classified data significantly increased in both the training and the test sets, concomitantly to a neat reduction of FP and FN signals (Table 3). On the contrary, the negligible fraction of UD spots (from 0.54% to 2.15%) was not significantly affected by the stepwise filtering process and should be assumed as a limitation of this genotyping microarray in its present form. In any case, after the stepwise filtering protocol FP signals remained the main source of errors in both the training (2.19%) and the test (3.84 to 6.87%) sets (Table 3). The main reason for this is that only perfect probe-target complementarity had been assigned to ‘expected hybridizations’ in the theoretical hybridization tables, assuming that any single mismatch between probe and target must always produce a negative hybridization signal. The experimental hybridization of a large number of microarrays has shown that this rather conservative criterion does not take into account that partial, though stable, hybridizations (in particular, when point mutations affect the 5’ or 3’ ends of the hybridizing sequences) are relatively frequent, regardless of the fact that the washing step had been optimized for increasing the overall performance of this genotyping microarray.

In turn, a fraction of the detected FN signals is due to the limitation that some target samples show a number of mutant nucleotides at the vicinity of the queried codon, thus preventing the correct hybridization to its corresponding and adjacent probes. This is exemplified by the target pINS of the training set: a TP signal is consistently obtained at probe Ins69c, while probes designed to cover positions 67, 68, 69 and 70 cannot hybridize to this target due to the silent mutations that accompany the genotype T69SSS [47,48]. Certain regions of the PR (e.g., at codons 46–48 and 82–90) and the RT (e.g., codons 67–70 and 210–215) are prone to accumulate a number of accompanying point mutations. Additionally, the sequences covered by contiguous probes often overlap, in such a way that a mutation in the target sample could affect its hybridization to probes adjacent to the one containing the queried codon. This is the case in codons 46–50 of the PR, as well as 65–70, 100–106, 179–184 and 210–215 of the RT. These facts limit the hybridization options in the neighbouring probe molecules even after the sequential optimization of the number and sequence of the spotted probes along the different versions of the microarray. Such effects, inherent to the genotyping of HIV-1 resistance mutations, are of special relevance in the cohort of clinical samples chosen for the test set, since they have been obtained from multi-treated patients that had received a number of complex drug combinations and thus showed numerous resistance mutations and accompanying silent mutations at the time of the sampling. However, the stepwise filtering protocol applied reduced the FN signals to values of 1.07% and 2.16–2.26% in the training and test sets, respectively (Table 3).

During the quality control protocol performed, 30 out of the 154 spotted probes were discarded. Interestingly, the vast majority of the discarded probes corresponded to redundant versions of some of them, which were designed to hybridize to the queried codons in different sequence contexts. Thus, probes complementary to the key HIV-1 codons involved in antiviral resistance and multidrug-resistance to NRTI and NNRTI (including different nucleotide insertions between RT codons 69 and 70, and the genotype associated with the Q151M complex) [7] were maintained after the filtering process (Tables A and D in S1 File). Concerning PIs, the only relevant probes that were discarded (and that should be redesigned in a further version of the microarray) correspond to the variants L90 and M90 of the PR.

The filtering protocol performed was also useful for identifying individual probes that compromise the overall accuracy of the genotyping microarray, thus being clear candidates to be redesigned in further versions of this genotyping platform. As an example, the NNRTI resistance-related probe Y181 (with an overlap between its positive and negative curves of 13.5%, see Figure G in S1 File) showed a high proportion of FN signals with samples from the training set. By discarding this probe in the analysis, the percentage of correctly classified spots would increase from 93.33% to 97.21% (remaining 1.77% of FP, 0.75% of FN and 0.26% of UD) and the overall performance of the genotyping microarray in the RT region would not significantly change (89.50% of correct, 6.56% of FP, 2.28% of FN and 1.65% of UD spots). In turn, seven of the probes that were discarded during the filtering protocol are relevant in the field of HIV-1 drug resistance and should be redesigned and tested for their inclusion in a further version of the microarray: M36-PR, A71PR-2, L90a-PR, K101-3, V179-2, L210-3 and K219. Moreover, new variants of some of the queried codons (e.g., 48-PR, 54-PR, 82-PR, 74, 75, 77, 103, 106, 179, 181, 190 and 219), as well as wild type and mutant probes for codons not included yet (e.g., 32-PR, 47-PR, 58-PR, 74-PR, 76-PR, 83-PR, 88-PR, 43, 98, 115, 138, 221 and 227), must be considered in the near future for increasing the clinical usefulness of the open platform provided by this genotyping microarray. Among the later, further versions of the microarray should include additional resistance mutations against new generation of PIs, in particular, mutations at PR codons 47 and 76 that confer resistance to Lopinavir and Darunavir [7] (Table D in S1 File). Tipranavir-associated resistance mutations could also be considered, though they are less relevant due to the limited use of this antiviral drug in clinical practice. In turn, integrase inhibitor resistance mutations associated with virological failure [7] should be included in further versions of the genotyping microarray, by selecting the corresponding codons along the IN region of the HIV-1 *pol* gene. Also, a panel of relevant resistance mutations corresponding to non-B subtype HIV-1 strains could be considered, thus improving the clinical applicability of this genotyping platform. Finally, the overall performance of the pre-hybridization steps should be further optimized to increase the PCR-based amplification efficiency for samples showing viral loads lower than 10^4^ HIV-1 RNA copies/ml.

In the current situation of antiretroviral treatment, emergence of HIV-1 drug resistance has strongly decreased in developed countries due to the potency of the new generation of drugs [6,7]. Also, easier treatment administration, lower toxicity and reduced side-effects result in an overall better adherence to combined antiretroviral therapies [66]. However, testing the potential drug resistance to the administered antiretroviral regimen is mandatory in case of viral load rebound [67]. Another situation in which resistance testing should be always performed corresponds to naïve patients before starting antiretroviral treatment. Although transmission of resistant HIV-1 variants is highly variable depending on the countries analyzed, different studies performed in Europe over the last decade suggest a relatively stable general prevalence of 8% to 10% for the transmission of viruses that are resistant to at least one antiretroviral drug [68–70]. Also, drug-resistant HIV-1 minority variants can be transmitted [71,72]. Of note, transmission of viruses carrying resistance mutations against NNRTIs is particularly relevant due to its prevalence (from 2% to 5%) and the high resistance level conferred by single mutations [73–75]. Transmission of PI resistance shows much lower prevalence (below 2%), while that of INI-resistance has only been communicated in isolated cases and has not been found in systematic surveys in Europe so far [76]. In this scenario, the developed genotyping microarray (in which drug resistance to both NRTIs and NNRTIs is detected with high sensitivity) would be particularly useful as a novel screening assay to genotype naïve patients before starting antiretroviral treatments.

## Conclusions

We have developed and tested an efficient, robust platform able to detect and quantify relevant mutations and other genotypic rearrangements that confer drug- or multidrug-resistance to NRTIs, NNRTIs and PIs, in both majority and minority (down to 5%) HIV-1 subpopulations. The microarray has been designed as an open, flexible genotyping platform that can be updated to include different spotted oligonucleotide probes containing newly detected codon mutations, insertions or deletions involved in drug resistance. It allows the high-throughput genotyping of up to 12 clinical samples in a working day, without requiring skilled technical personnel along the experimental work-flow. The current protocol is adapted to the use of at least two different types of microarray scanners to get the hybridization signals. The developed bioinformatics algorithm provides a highly accurate profiling of filtered hybridization signals of relevant majority and minority drug-resistance variants. Thus, this genotyping microarray is proposed as an open and customizable platform useful for drug-resistance diagnosis and prognosis in clinical practice focused on personalized antiretroviral therapies.

## Supporting Information

S1 File Figures A-L and Tables A-D.
(PDF)
