# Supplementary material for: An Efficient Microarray-Based Genotyping Platform for the Identification of Drug-Resistance Mutations in Majority and Minority Subpopulations of HIV-1 Quasispecies
Source: PLoS One. 2016 Dec 13;11(12):e0166902. doi: 10.1371/journal.pone.0166902 (PMC5154500; doi:10.1371/journal.pone.0166902)
Supplement: S1 File — Figures A-L and Tables A-D. (PDF) [file pone.0166902.s001.pdf]

# An efficient microarray-based genotyping platform for the identification of drug-resistance mutations in majority and minority subpopulations of HIV-1 quasispecies

## *S1 File: Supporting information*

Verónica Martín[1,#a], Celia Perales[1,2,3], María Fernández-Algar[4], Helena G. Dos Santos[1,#b], Patricia Garrido[5,#c], María Pernas[5,#d], Víctor Parro[4], Miguel Moreno[4], Javier García-Pérez[6], José Alcamí[6], José Luis Torán[5,#e], David Abia[1], Esteban Domingo[1,2], Carlos Briones[2,4,]*.

[1] Centro de Biología Molecular 'Severo Ochoa' (CBMSO, CSIC-UAM). Campus de Cantoblanco, 28049 Madrid, Spain.

[2] Centro de Investigación Biomédica en Red de enfermedades hepáticas y digestivas (CIBERehd), Spain.

[3] Liver Unit, Internal Medicine, Laboratory of Malalties Hepàtiques, Vall d'Hebron Institut de Recerca-Hospital Universitari Vall d´Hebron (VHIR-HUVH), Universitat Autònoma de Barcelona. 08035 Barcelona, Spain.

[4] Department of Molecular Evolution, Centro de Astrobiología (CAB, CSIC-INTA). 28850 Torrejón de Ardoz, Madrid, Spain.

[5] Biotherapix, SLU. Parque Tecnológico de Madrid, 28760 Tres Cantos, Madrid. Spain.

[6] AIDS Immunopathogenesis Unit, Instituto de Salud Carlos III. 28220 Majadahonda, Madrid, Spain.

[#a]Current Address: Centro de Investigación en Sanidad Animal (CISA), Instituto Nacional de Investigación y Tecnología Agraria y Alimentaria (INIA). 28130 Valdeolmos, Madrid, Spain.

[#b]Current Address: Department of Biological Sciences, Florida International University. Miami, FL 33199, USA.

[#c]Current Address: Sygnis Biotech. Parque Científico de Madrid. Campus de Cantoblanco, 28049 Madrid, Spain.

[#d]Current Address: Instituto de Salud Carlos III. 28220 Majadahonda, Madrid, Spain.

[#e]Current Address: Spanish National Center for Biotechnology (CNB, CSIC). Campus de Cantoblanco, 28049 Madrid, Spain.

* Corresponding author

E-mail: cbriones@cab.inta-csic.es

**Figure A. Scheme of the HIV-1 genotyping microarray and examples of the hybridization signals produced by wild type target DNA molecules.** (a) Position of the 360 spots printed on each of the four subarrays of the microarray. Discriminating probes are shown in black, positive HIV-1 internal hybridization controls (IHC-PR3, IHC-PR4, IHC-RT3 and IHC-RT4) are depicted in green, negative controls (C-:FMDV-G142-15r and C-:FMDV-E142-15r, corresponding to sequences of the foot-and-mouth disease virus VP1 coding region) appear in red and additional negative controls including spotting solution (C-) are shown in grey. Yellow background highlights the probes complementary to wild type sequences for each queried codon. The highly performing probes that were maintained after the quality control protocol (see main text) are underlined. Further details on the probe sequences are given in Supp. Table S1. Rows 1-4 contain the PR-specific probes and rows 5-15 the RT ones. (b) and (c) Examples of microarray images corresponding to the hybridization of PR and RT targets with wild type sequences, using ScanArray and GenePix microarray scanners (see Methods), respectively.

a)

| | A | B | C | D | E | F | G | H | I | J | K | L |
|---|---|---|---|---|---|---|---|---|---|---|---|---|
| 1 | IHC-PR3 | IHC-PR4 | R8-PR-2 | Q8-PR-2 | D30-PR | N30-PR | M36-PR | I36-PR | M46b-PR | M46c-PR | F46-PR | I46-PR |
| 2 | L46-PR | V46-PR | G48-PR | V48-PR | I50-PR | L50a-PR | L50b-PR | V50-PR-2 | I54-PR | V54-PR-2 | A71-PR-2 | V71-PR-2 |
| 3 | V82b-PR-2 | A82-PR-2 | F82-PR-2 | I82-PR-2 | S82-PR-2 | T82-PR-2 | I84-PR-2 | A84-PR-2 | V84-PR-2 | L90a-PR | L90a-PR-2 | I90-PR |
| 4 | I90-PR-2 | M90-PR | M90-PR-2 | C-: FMDV-G142-15r | C-: FMDV-E142-15r | C- | C- | C- | C- | C- | IHC-PR3 | IHC-PR4 |
| 5 | IHC-RT3 | IHC-RT4 | M41 | L41a | L41b | A62 | V62 | K65-2 | R65-3 | D67a-2 | D67b-2 | E67-2 |
| 6 | G67 | G67-2 | N67-2 | Del67 | S68 | N68 | T69b | T69c | A69 | D69 | G69 | S69a |
| 7 | S69b | S69R70 | Ins69a | Ins69b | Ins69c | Ins69d | Ins69e | Ins69f | Ins69g | Ins69h | Ins69i | Ins69j |
| 8 | Ins69k | Ins69l | K70a | K70b | E70 | N70a | N70b | R70a | R70b | L74 | V74-2 | V75 |
| 9 | I75 | T75 | F77 | L77 | L100-2 | I100-2 | K101-3 | E101-3 | K103a-2 | K103c-2 | N103-3 | R103-3 |
| 10 | V106-2 | A106-2 | I106-2 | L106-2 | V108-2 | I108-2 | F116 | Y116-3 | Q151-2 | M151-2 | I178 | M178 |
| 11 | V179-2 | D179-2 | E179-2 | Y181 | C181 | H181 | I181 | L181 | M184a | M184b | I184 | T184 |
| 12 | V184b | Y188a | Y188c | C188-2 | H188 | L188a | L188b | G190 | A190 | E190 | Q190 | S190 |
| 13 | T190 | L210-3 | W210-2 | R211-2 | K211-2 | T215a-2 | T215b-2 | C215-2 | F215-2 | S215-2 | Y215-2 | K219 |
| 14 | K219-2 | E219 | Q219 | P225-2 | H225-2 | M230-3 | L230-3 | P236-2 | L236-2 | K238 | T238 | C-: FMDV-G142-15r |
| 15 | C-: FMDV-E142-15r | C- | C- | C- | C- | C- | C- | C- | C- | C- | IHC-RT3 | IHC-RT4 |

b)

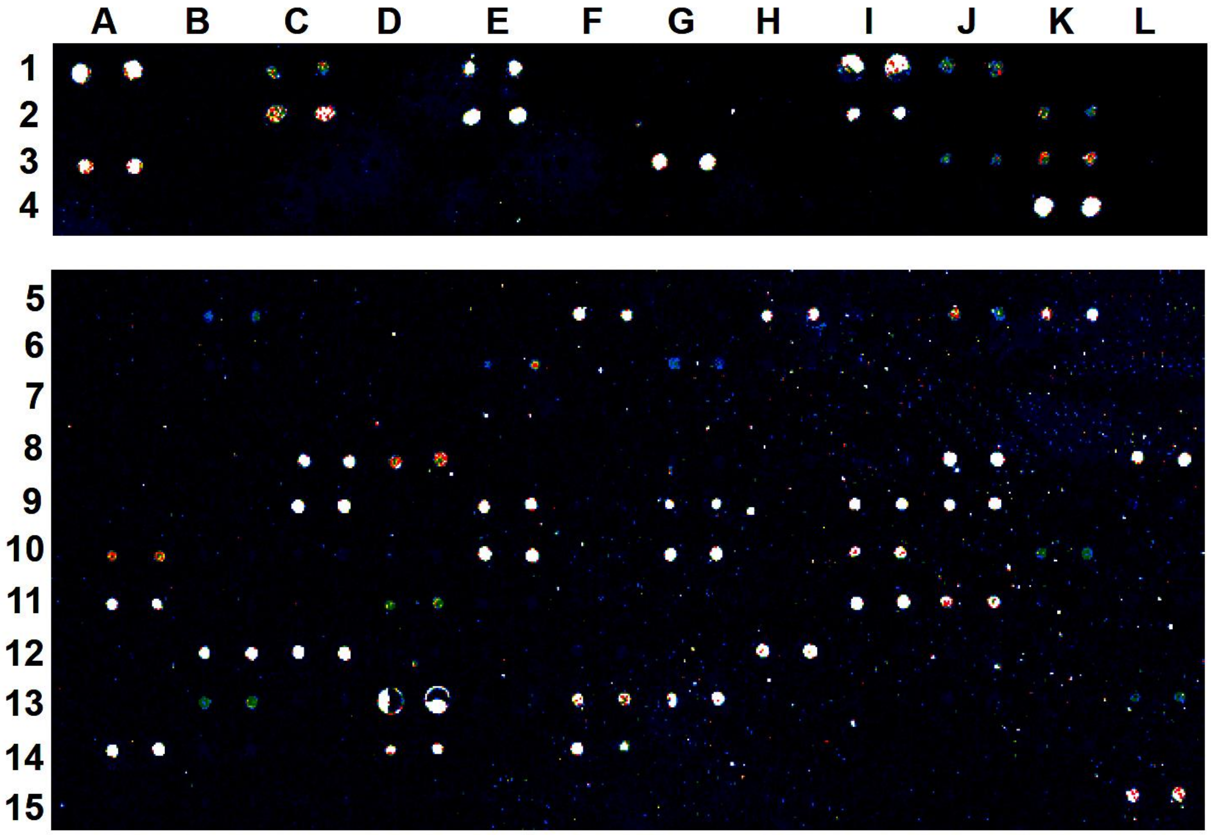
A B C D E F G H I J K L
1 2 3 4 5 6 7 8 9 10 11 12 13 14 15

c)

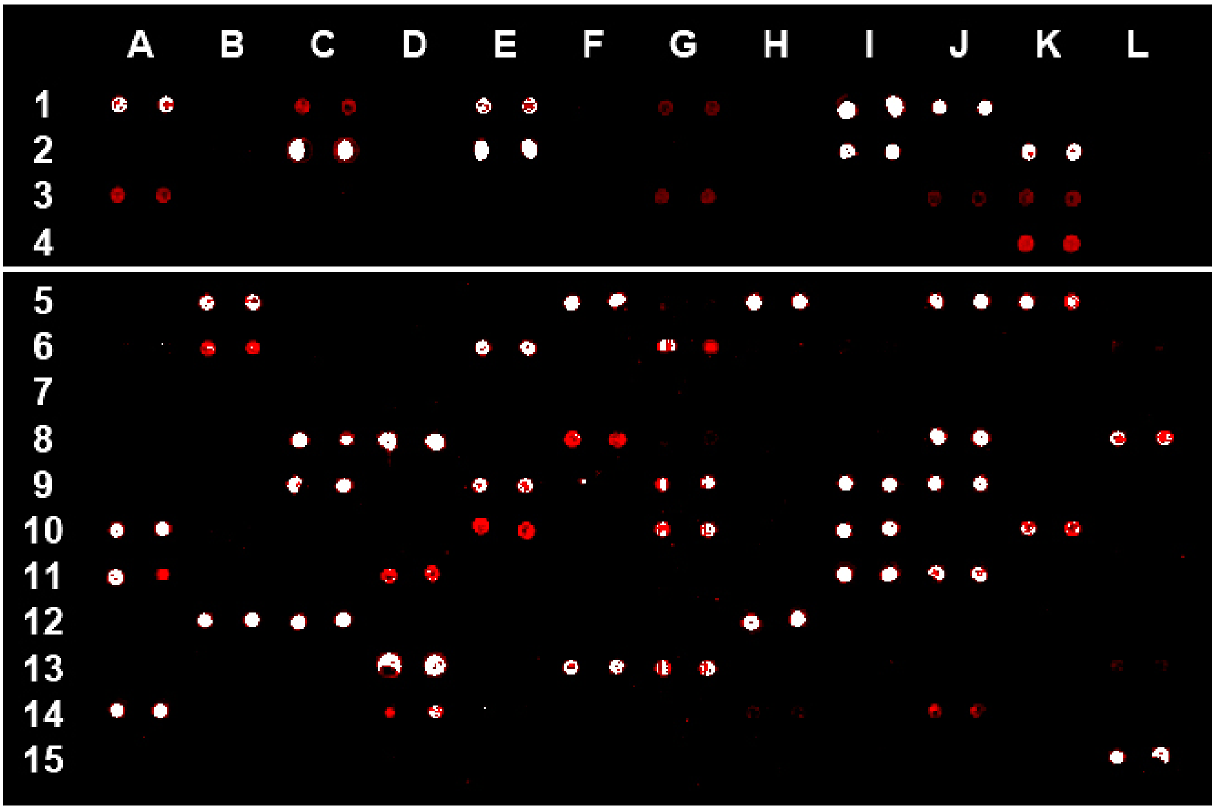
A B C D E F G H I J K L
1 2 3 4 5 6 7 8 9 10 11 12 13 14 15

**Figure B. Examples of the hybridization results of different PR and RT targets including drug resistance mutations and insertions.** (a) Discrimination of mutants in the HIV-1 PR: I36, I46, L46, V48, V54, V71, T82, V84, M90+M90-2 (highlighted in red). (b) Discrimination of mutant and insert-containing targets of the HIV-1 RT: V62, S68, S69, Ins69a, Ins69b, Ins69c, Ins69d, I75, T75, I100, E101, I108, M151, M178, C181, I184, H188, K211, Y215, C215 and T238. The whole set of raw microarray hybridization data used in this study has been uploaded to Gene Expression Omnibus (GEO) repository (https://www.ncbi.nlm.nih.gov/geo/), with the accession number GSE90621.

a)

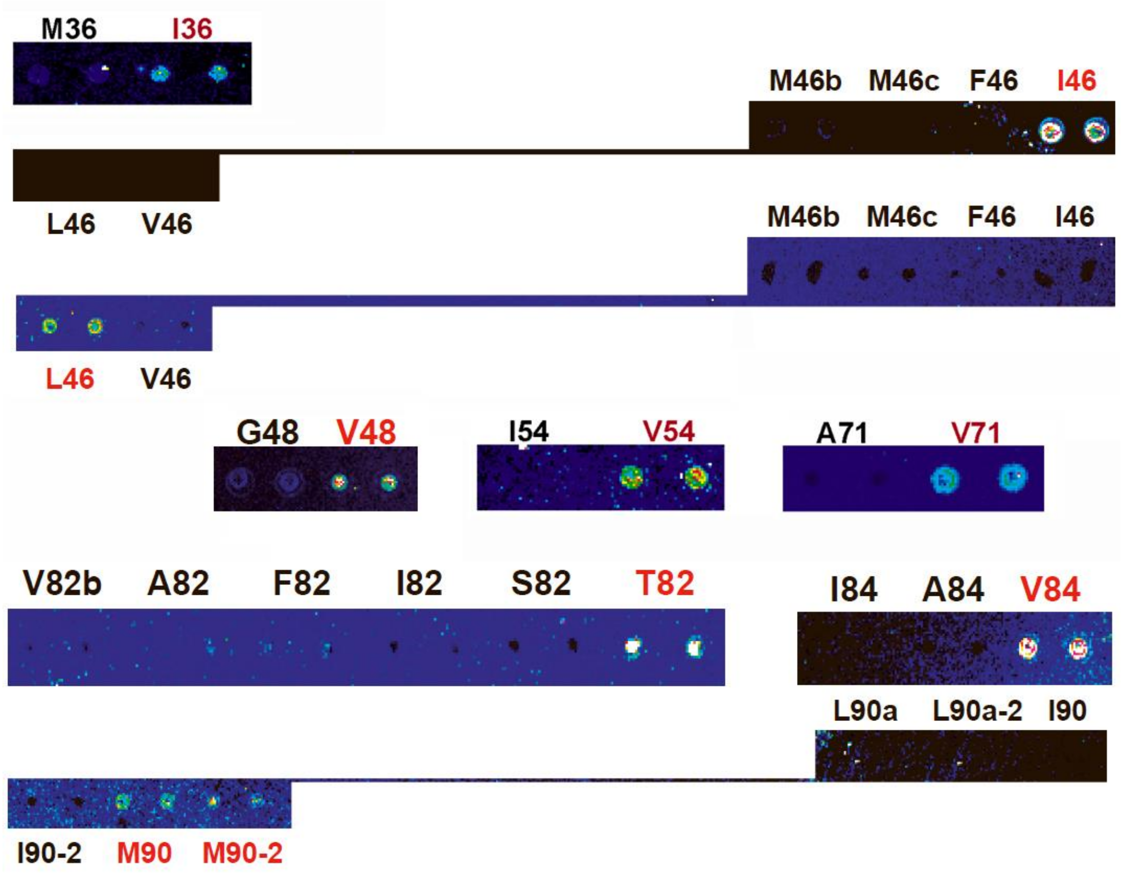

b)

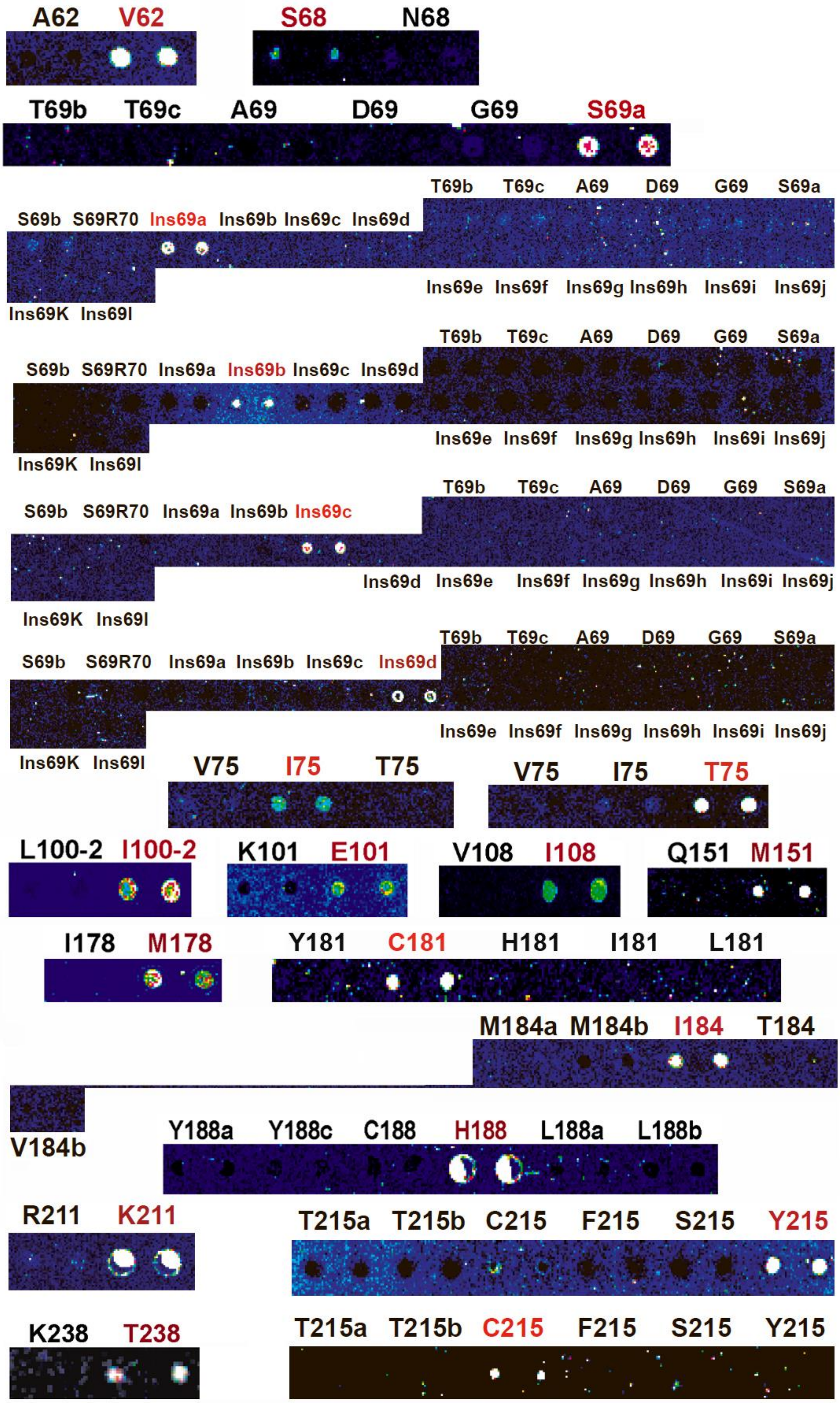
A62 V62
S68 N68
T69b T69c A69 D69 G69 S69a
T69b T69c A69 D69 G69 S69a
S69b S69R70 Ins69a Ins69b Ins69c Ins69d
Ins69e Ins69f Ins69g Ins69h Ins69i Ins69j
Ins69K Ins69l
T69b T69c A69 D69 G69 S69a
S69b S69R70 Ins69a Ins69b Ins69c Ins69d
Ins69e Ins69f Ins69g Ins69h Ins69i Ins69j
Ins69K Ins69l
T69b T69c A69 D69 G69 S69a
S69b S69R70 Ins69a Ins69b Ins69c
Ins69d Ins69e Ins69f Ins69g Ins69h Ins69i Ins69j
Ins69K Ins69l
T69b T69c A69 D69 G69 S69a
S69b S69R70 Ins69a Ins69b Ins69c Ins69d
Ins69e Ins69f Ins69g Ins69h Ins69i Ins69j
Ins69K Ins69l
V75 I75 T75
V75 I75 T75
L100-2 I100-2
K101 E101
V108 I108
Q151 M151
I178 M178
Y181 C181 H181 I181 L181
M184a M184b I184 T184
V184b
Y188a Y188c C188 H188 L188a L188b
R211 K211
T215a T215b C215 F215 S215 Y215
K238 T238
T215a T215b C215 F215 S215 Y215

**Figure C. Examples of the hybridization results produced by different RT targets belonging to the clinical samples of the test set that contained majority and minority variants.** Positive signals in the microarray are highlighted in red. The theoretical percentage of signals at each probe position, derived from the analysis of clonal sequences (13 to 34 clones per sample) is shown below the hybridization signals. Different situations have been chosen to exemplify either concordant or discrepant results between the hybridization signals and their expected percentage derived from clonal analysis (discrepant percentages are depicted in grey). The sequencing electropherograms corresponding to the population sequence are also shown, together with the derived amino acid(s) assumed to be present at the interrogating codon.

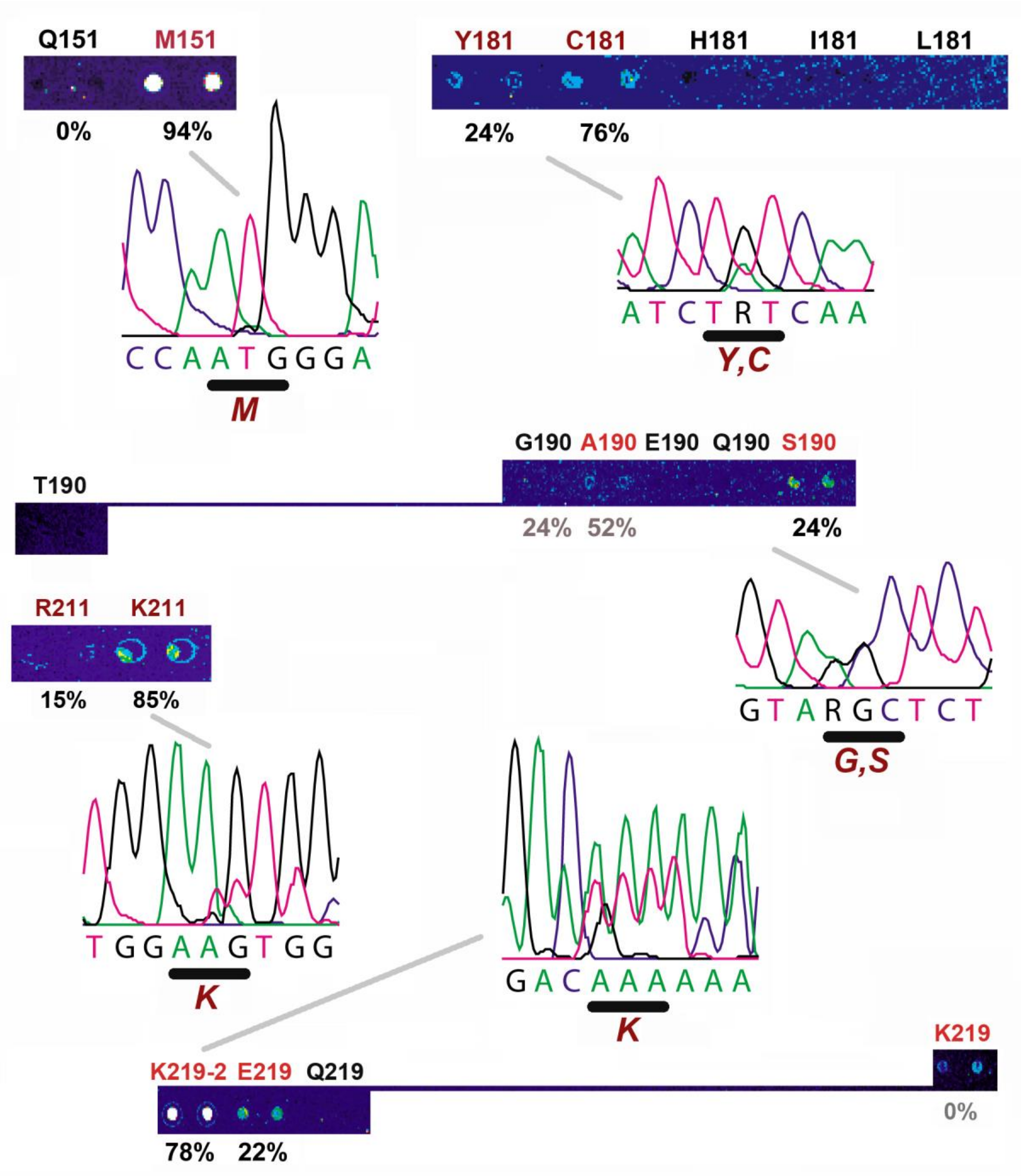

**Figure D. Theoretical hybridization tables of the training set.** Tables show the sequences of the pure clonal samples (rows in panel a, columns in b) that are complementary to the sequences of the spotted probes (columns and rows, respectively) belonging to the PR (a) and RT (b) regions. Color code: Green, expected hybridization; Red, not expected hybridization; Black, partial hybridization (one mismatch between probe and target is present at the 5’ or 3’ position of the hybridizing sequence).

a)

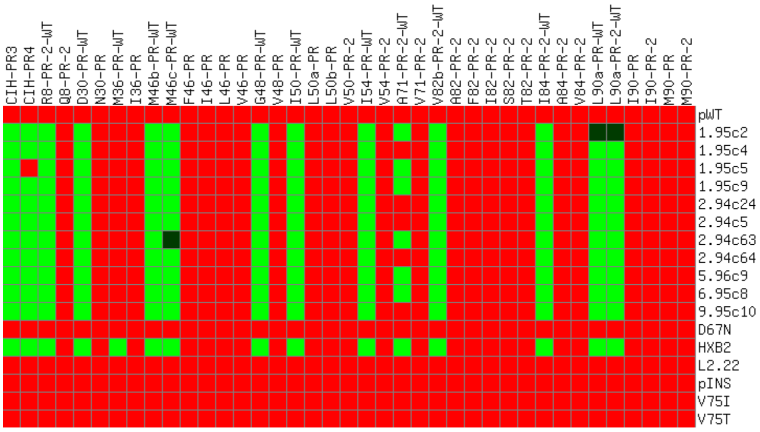

b)

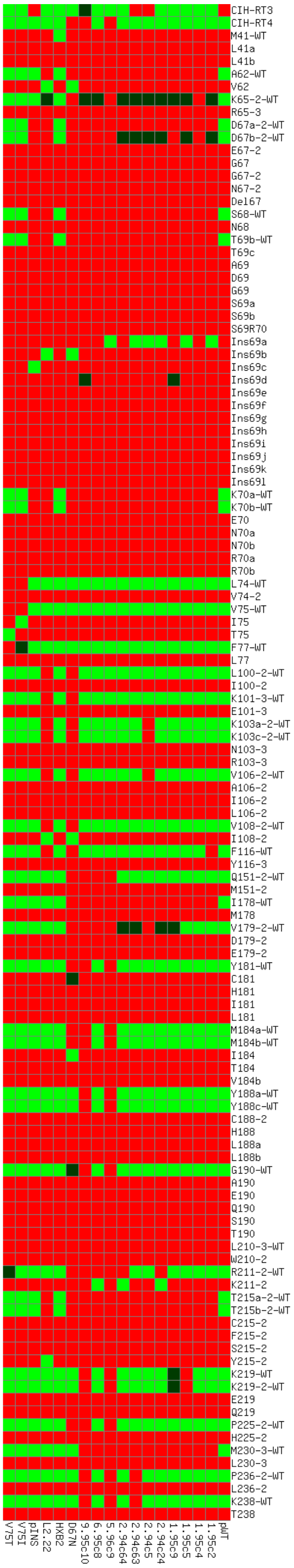

CIH-RT3
CIH-RT4
M41-WT
L41a
L41b
A62-WT
V62
K65-2-WT
R65-3
D67a-2-WT
D67b-2-WT
E67-2
G67
G67-2
N67-2
Del67
S68-WT
N68
T69b-WT
T69c
A69
D69
G69
S69a
S69b
S69R70
Ins69a
Ins69b
Ins69c
Ins69d
Ins69e
Ins69f
Ins69g
Ins69h
Ins69i
Ins69j
Ins69k
Ins69l
K70a-WT
K70b-WT
E70
N70a
N70b
R70a
R70b
L74-WT
V74-2
V75-WT
I75
T75
F77-WT
L77
L100-2-WT
I100-2
K101-3-WT
E101-3
K103a-2-WT
K103c-2-WT
N103-3
R103-3
V106-2-WT
A106-2
I106-2
L106-2
V108-2-WT
I108-2
F116-WT
Y116-3
Q151-2-WT
M151-2
I178-WT
M178
V179-2-WT
D179-2
E179-2
Y181-WT
C181
H181
I181
L181
M184a-WT
M184b-WT
I184
T184
V184b
Y188a-WT
Y188c-WT
C188-2
H188
L188a
L188b
G190-WT
A190
E190
Q190
S190
T190
L210-3-WT
W210-2
R211-2-WT
K211-2
T215a-2-WT
T215b-2-WT
C215-2
F215-2
S215-2
Y215-2
K219-WT
K219-2-WT
E219
Q219
P225-2-WT
H225-2
M230-3-WT
L230-3
P236-2-WT
L236-2
K238-WT
T238
V75T
V75I
pINS
L2.22
HXB2
D67N
9.95c10
6.95c8
5.96c9
2.94c64
2.94c63
2.94c5
2.94c24
1.95c9
1.95c5
1.95c4
1.95c2
pWT

**Figure E. Density of the raw hybridization data for three key variables quantified by *GenePix* and *ScanArray* scanners.** The threshold values are shown with dotted lines. Color code: Green, true positive (TP) signal; Red, false positive (FP); Blue, true negative (TN); Black, false negative (FN).

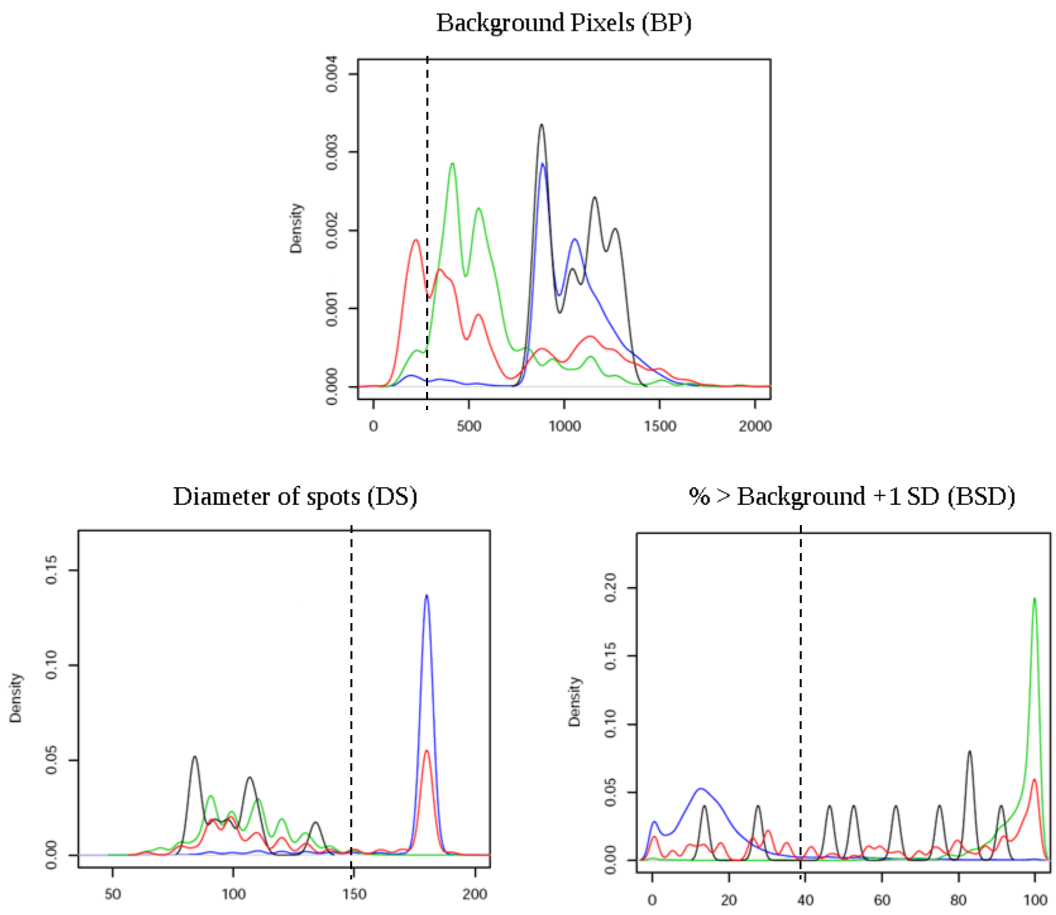

**Figure F. Test of the normalization performance of data from the training set.** a) Density of positive and negative raw fluorescence data. b) Normalized fluorescence data using the mean positive signal of each array region (RT or RT): distributions of positive signals are centred in value 1 of normalized intensity, while distributions of negative signals are close to zero. Color code: Red, density of negative hybridization data; Blue, fit to a log-normal distribution of negative data; Black, density of positive hybridization data; Green, fit to a normal distribution of positive data.

a)

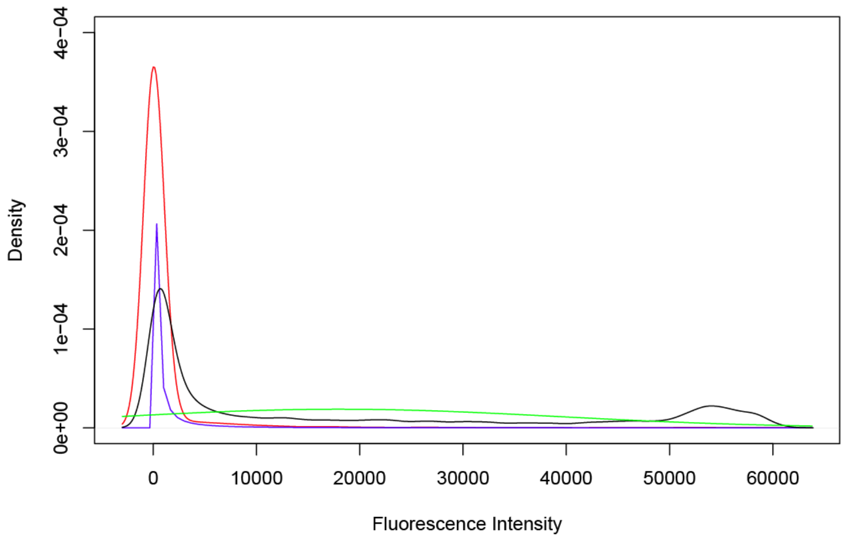


b)

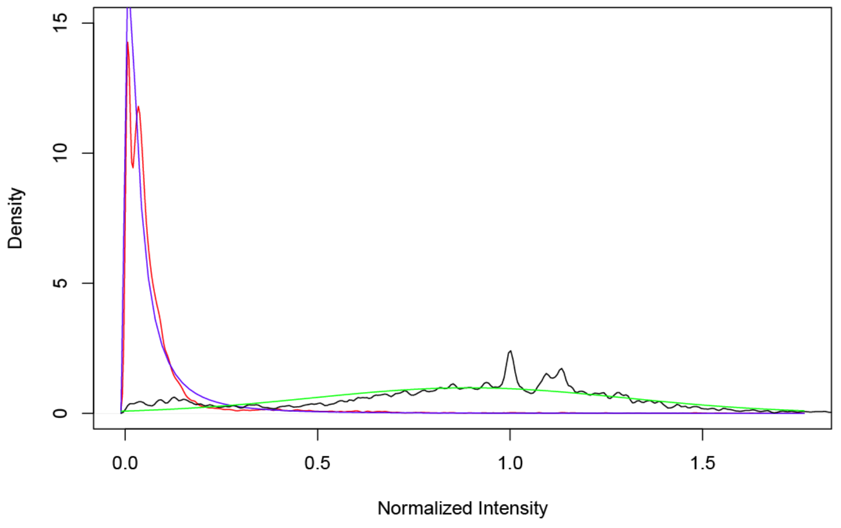

**Figure G. Probe-specific curves for the 124 spotted probes that passed the quality control.** Density of normalized hybridization signals from the training set with their fitted distribution functions for positive and negative data. Color code: see Supp. Fig S6.

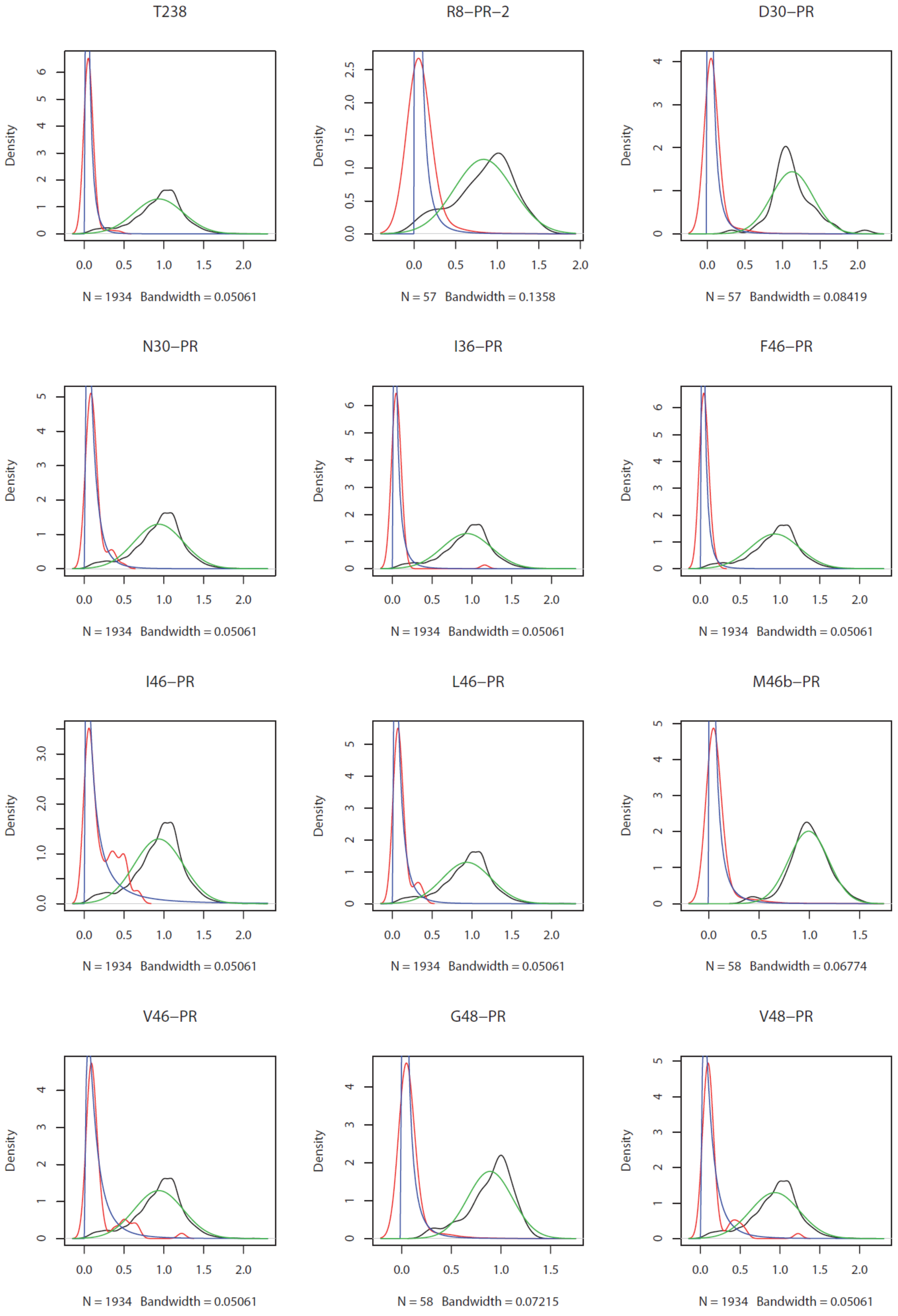

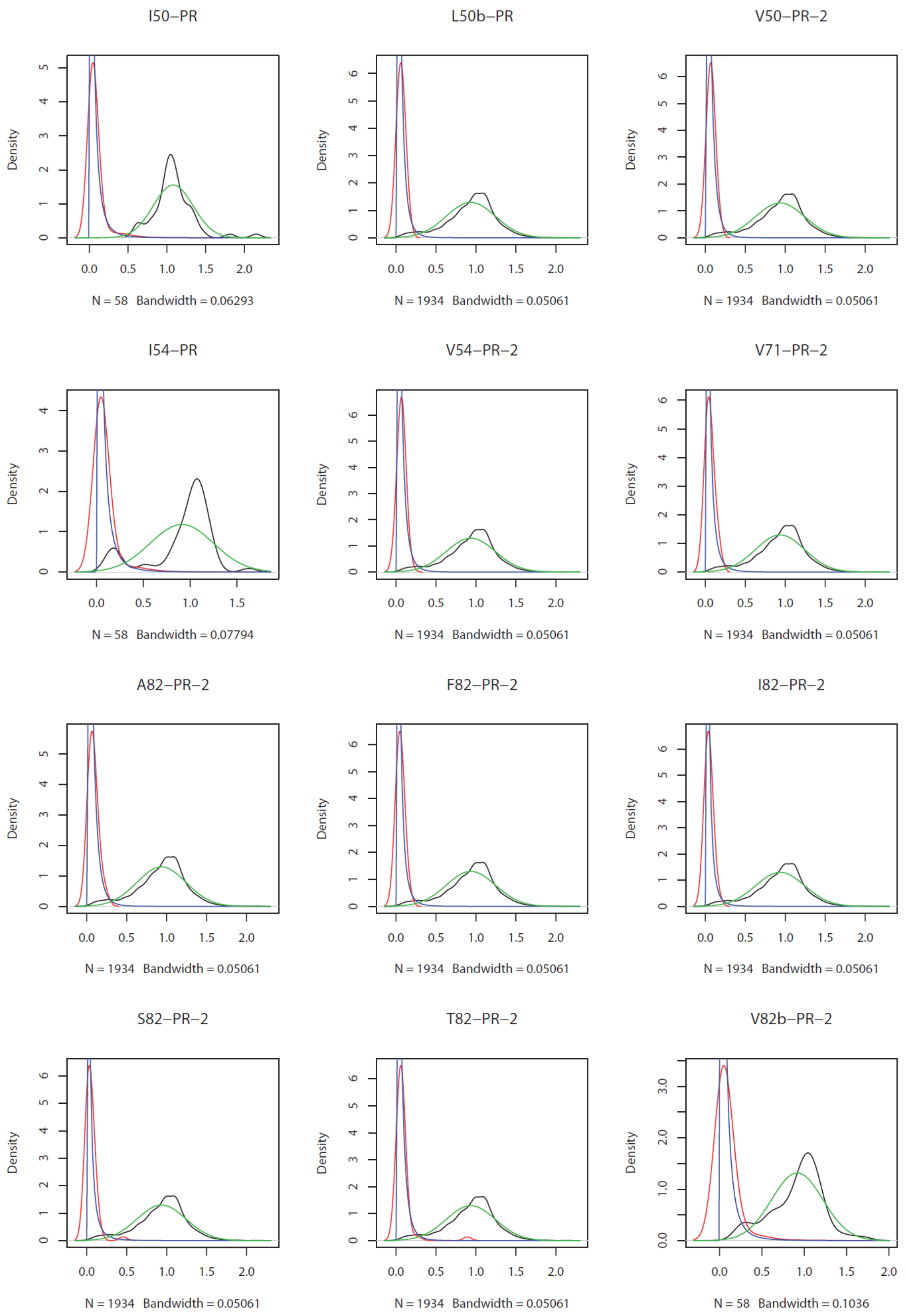
I50–PR
Density
N = 58 Bandwidth = 0.06293
L50b–PR
Density
N = 1934 Bandwidth = 0.05061
V50–PR–2
Density
N = 1934 Bandwidth = 0.05061
I54–PR
Density
N = 58 Bandwidth = 0.07794
V54–PR–2
Density
N = 1934 Bandwidth = 0.05061
V71–PR–2
Density
N = 1934 Bandwidth = 0.05061
A82–PR–2
Density
N = 1934 Bandwidth = 0.05061
F82–PR–2
Density
N = 1934 Bandwidth = 0.05061
I82–PR–2
Density
N = 1934 Bandwidth = 0.05061
S82–PR–2
Density
N = 1934 Bandwidth = 0.05061
T82–PR–2
Density
N = 1934 Bandwidth = 0.05061
V82b–PR–2
Density
N = 58 Bandwidth = 0.1036

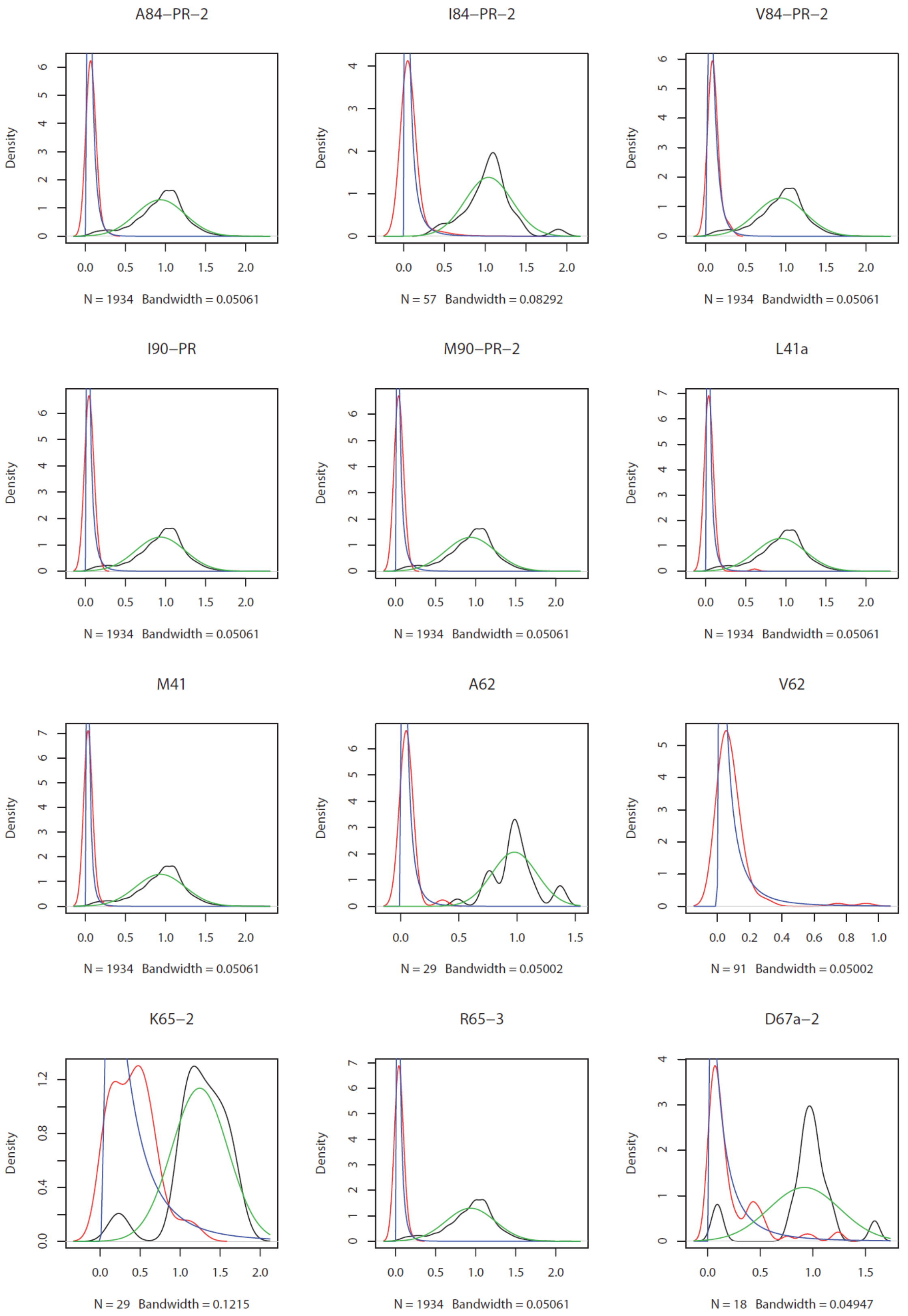
A84–PR–2
N = 1934 Bandwidth = 0.05061
I84–PR–2
N = 57 Bandwidth = 0.08292
V84–PR–2
N = 1934 Bandwidth = 0.05061
I90–PR
N = 1934 Bandwidth = 0.05061
M90–PR–2
N = 1934 Bandwidth = 0.05061
L41a
N = 1934 Bandwidth = 0.05061
M41
N = 1934 Bandwidth = 0.05061
A62
N = 29 Bandwidth = 0.05002
V62
N = 91 Bandwidth = 0.05002
K65–2
N = 29 Bandwidth = 0.1215
R65–3
N = 1934 Bandwidth = 0.05061
D67a–2
N = 18 Bandwidth = 0.04947
Density

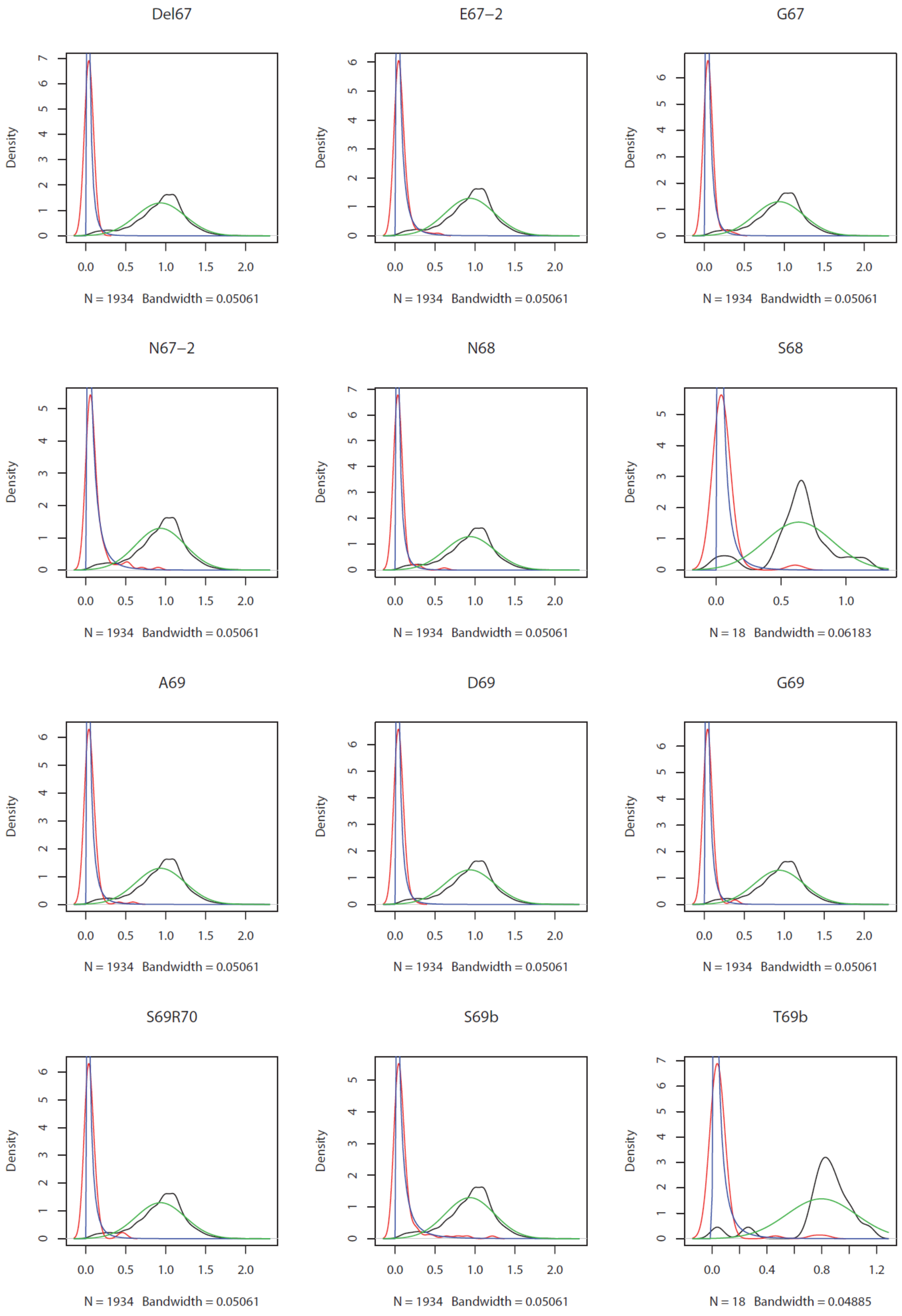

Del67
Density
N = 1934 Bandwidth = 0.05061
E67−2
Density
N = 1934 Bandwidth = 0.05061
G67
Density
N = 1934 Bandwidth = 0.05061
N67−2
Density
N = 1934 Bandwidth = 0.05061
N68
Density
N = 1934 Bandwidth = 0.05061
S68
Density
N = 18 Bandwidth = 0.06183
A69
Density
N = 1934 Bandwidth = 0.05061
D69
Density
N = 1934 Bandwidth = 0.05061
G69
Density
N = 1934 Bandwidth = 0.05061
S69R70
Density
N = 1934 Bandwidth = 0.05061
S69b
Density
N = 1934 Bandwidth = 0.05061
T69b
Density
N = 18 Bandwidth = 0.04885

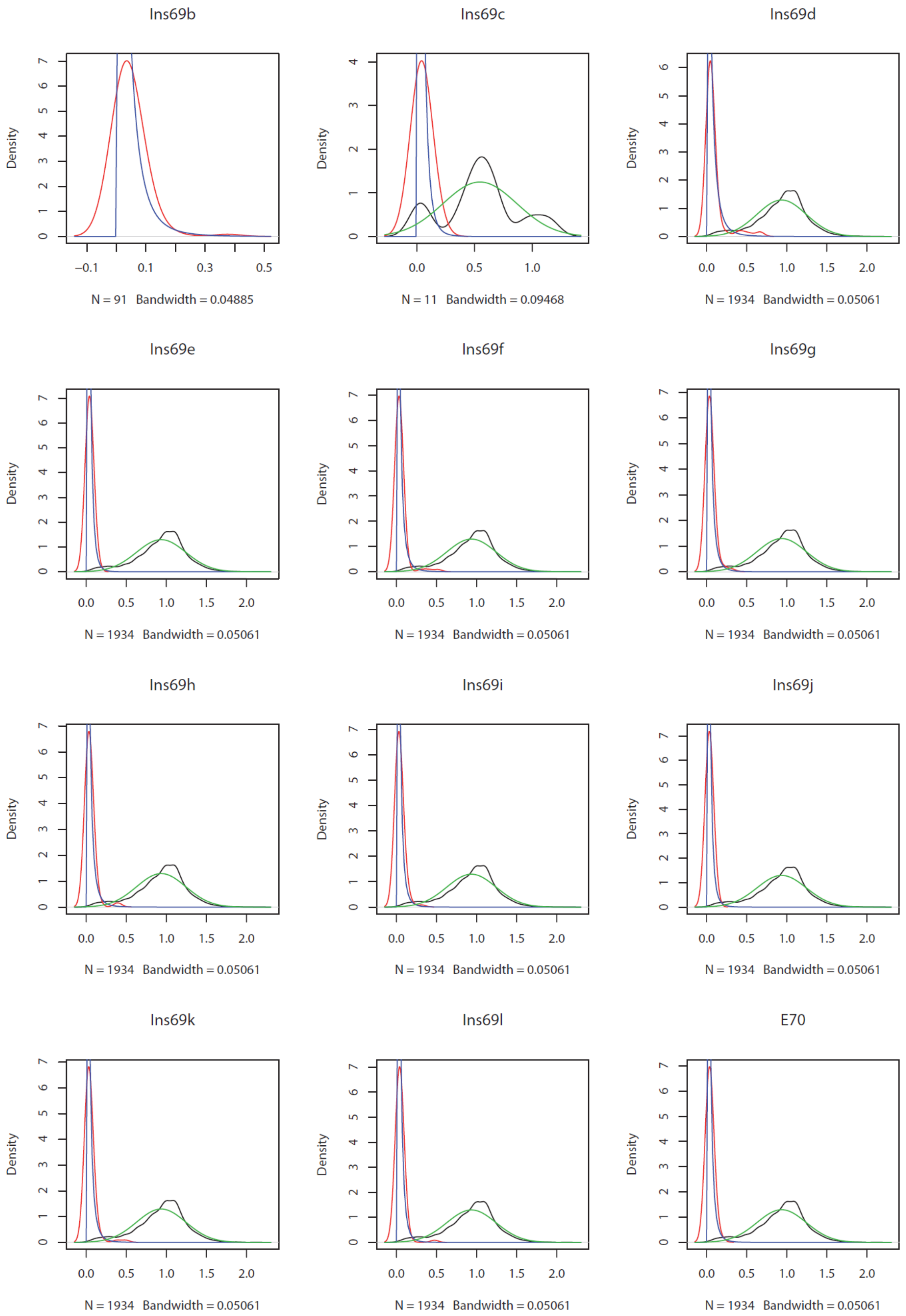
Ins69b
Density
−0.1 0.1 0.3 0.5
N = 91 Bandwidth = 0.04885
Ins69c
Density
0.0 0.5 1.0
N = 11 Bandwidth = 0.09468
Ins69d
Density
0.0 0.5 1.0 1.5 2.0
N = 1934 Bandwidth = 0.05061
Ins69e
Density
0.0 0.5 1.0 1.5 2.0
N = 1934 Bandwidth = 0.05061
Ins69f
Density
0.0 0.5 1.0 1.5 2.0
N = 1934 Bandwidth = 0.05061
Ins69g
Density
0.0 0.5 1.0 1.5 2.0
N = 1934 Bandwidth = 0.05061
Ins69h
Density
0.0 0.5 1.0 1.5 2.0
N = 1934 Bandwidth = 0.05061
Ins69i
Density
0.0 0.5 1.0 1.5 2.0
N = 1934 Bandwidth = 0.05061
Ins69j
Density
0.0 0.5 1.0 1.5 2.0
N = 1934 Bandwidth = 0.05061
Ins69k
Density
0.0 0.5 1.0 1.5 2.0
N = 1934 Bandwidth = 0.05061
Ins69l
Density
0.0 0.5 1.0 1.5 2.0
N = 1934 Bandwidth = 0.05061
E70
Density
0.0 0.5 1.0 1.5 2.0
N = 1934 Bandwidth = 0.05061

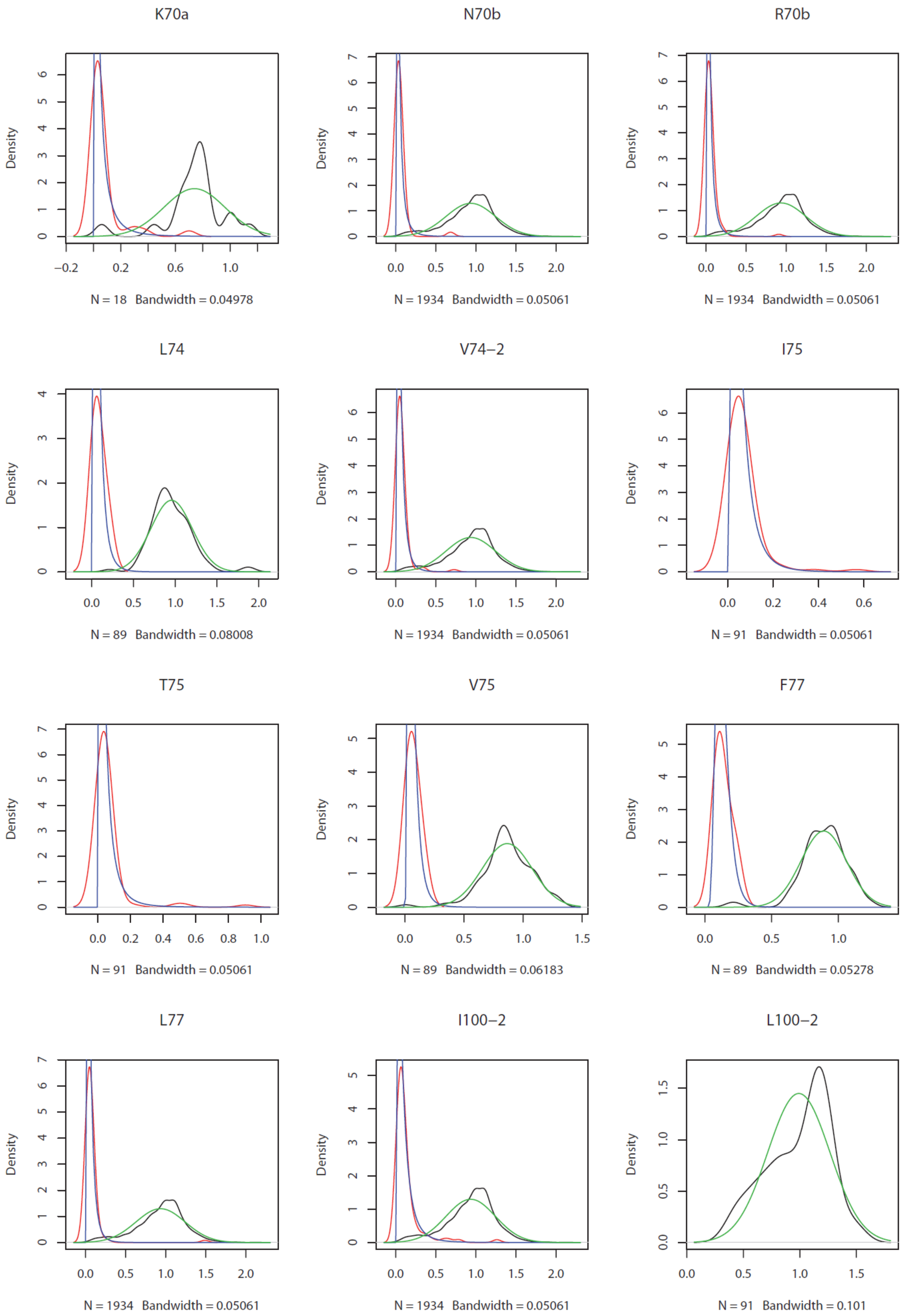
K70a
N = 18 Bandwidth = 0.04978
N70b
N = 1934 Bandwidth = 0.05061
R70b
N = 1934 Bandwidth = 0.05061
L74
N = 89 Bandwidth = 0.08008
V74–2
N = 1934 Bandwidth = 0.05061
I75
N = 91 Bandwidth = 0.05061
T75
N = 91 Bandwidth = 0.05061
V75
N = 89 Bandwidth = 0.06183
F77
N = 89 Bandwidth = 0.05278
L77
N = 1934 Bandwidth = 0.05061
I100–2
N = 1934 Bandwidth = 0.05061
L100–2
N = 91 Bandwidth = 0.101
Density

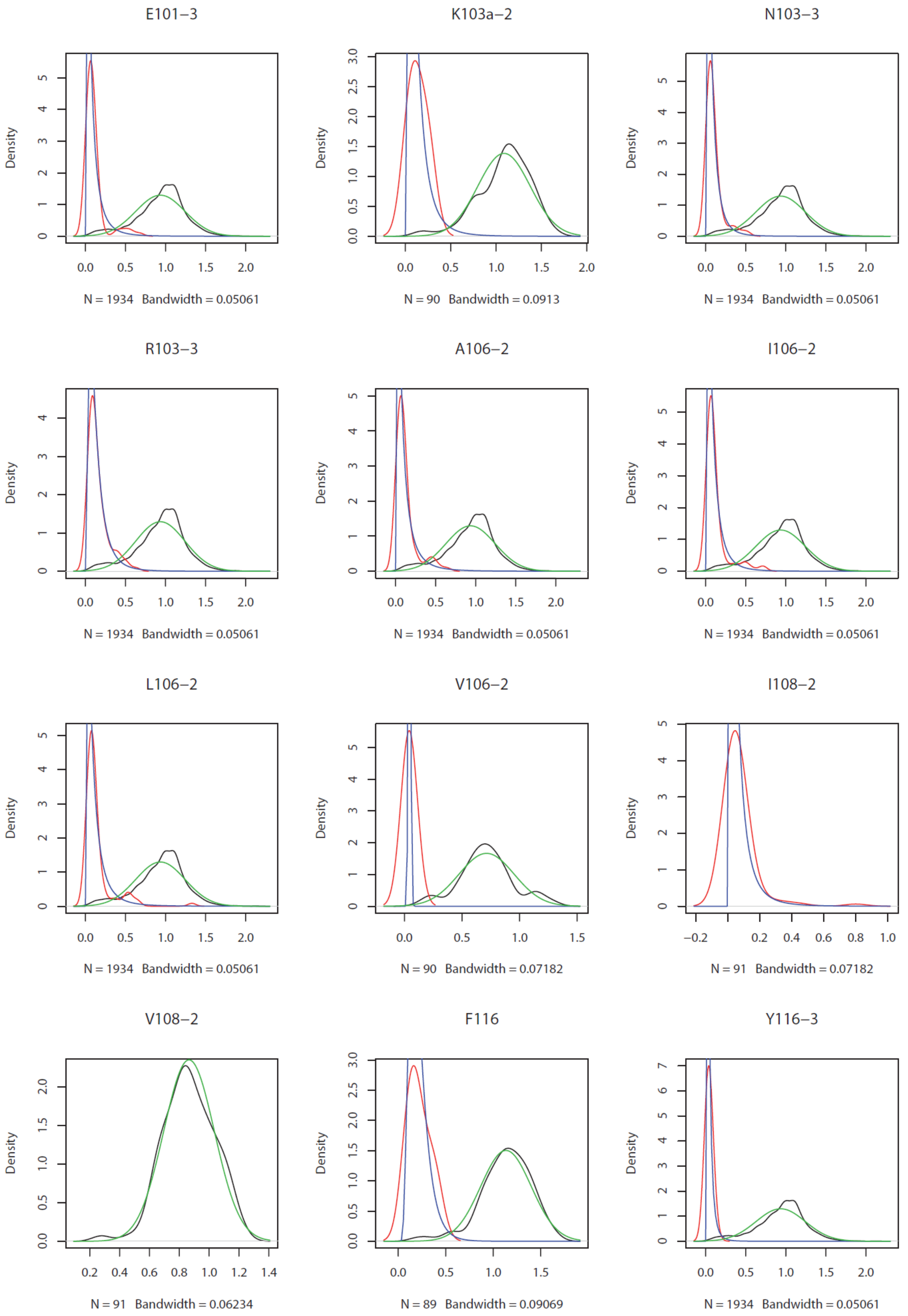

E101–3
Density
N = 1934 Bandwidth = 0.05061
K103a–2
Density
N = 90 Bandwidth = 0.0913
N103–3
Density
N = 1934 Bandwidth = 0.05061
R103–3
Density
N = 1934 Bandwidth = 0.05061
A106–2
Density
N = 1934 Bandwidth = 0.05061
I106–2
Density
N = 1934 Bandwidth = 0.05061
L106–2
Density
N = 1934 Bandwidth = 0.05061
V106–2
Density
N = 90 Bandwidth = 0.07182
I108–2
Density
N = 91 Bandwidth = 0.07182
V108–2
Density
N = 91 Bandwidth = 0.06234
F116
Density
N = 89 Bandwidth = 0.09069
Y116–3
Density
N = 1934 Bandwidth = 0.05061

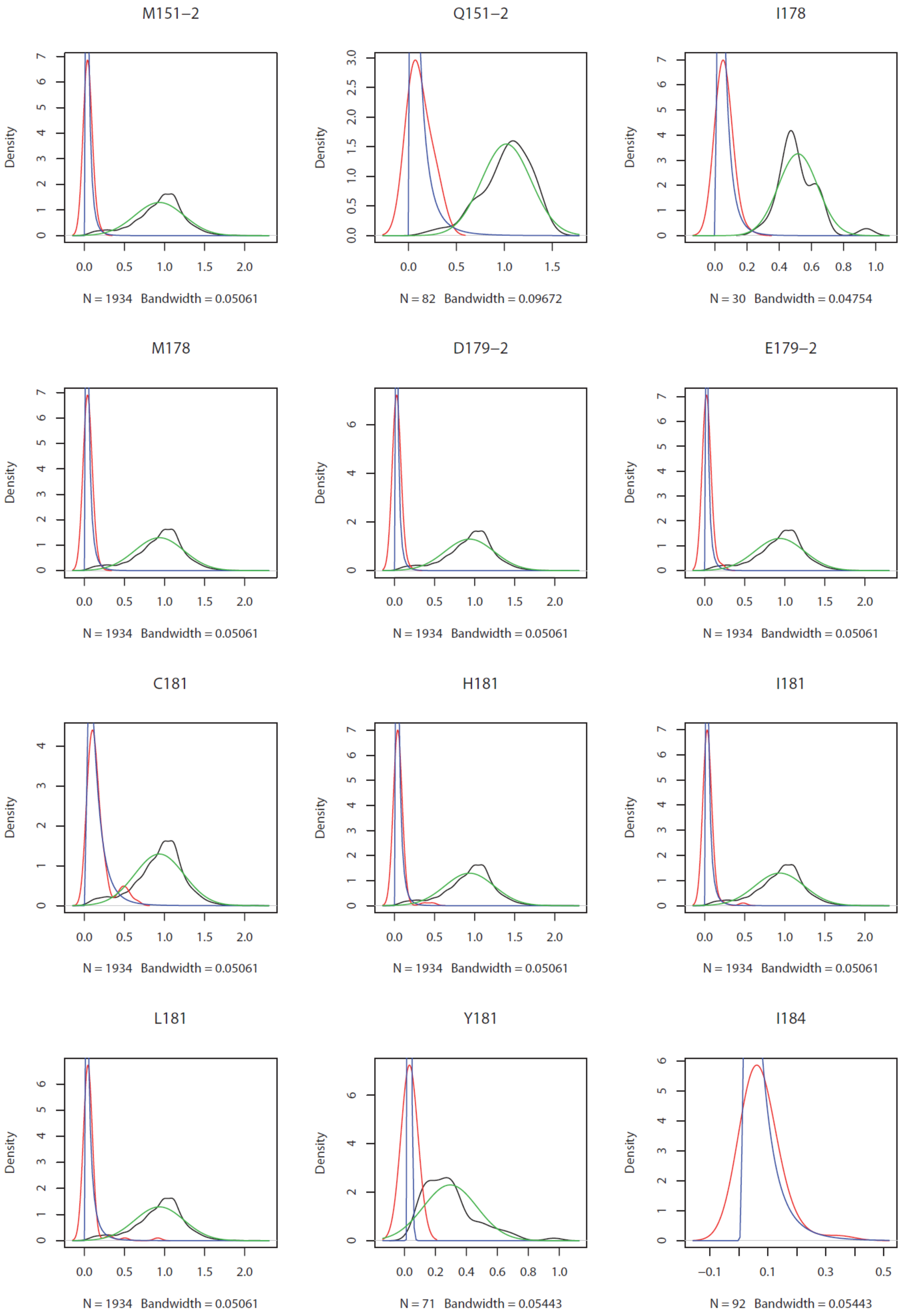

M151–2
Density
N = 1934 Bandwidth = 0.05061
Q151–2
Density
N = 82 Bandwidth = 0.09672
I178
Density
N = 30 Bandwidth = 0.04754
M178
Density
N = 1934 Bandwidth = 0.05061
D179–2
Density
N = 1934 Bandwidth = 0.05061
E179–2
Density
N = 1934 Bandwidth = 0.05061
C181
Density
N = 1934 Bandwidth = 0.05061
H181
Density
N = 1934 Bandwidth = 0.05061
I181
Density
N = 1934 Bandwidth = 0.05061
L181
Density
N = 1934 Bandwidth = 0.05061
Y181
Density
N = 71 Bandwidth = 0.05443
I184
Density
N = 92 Bandwidth = 0.05443

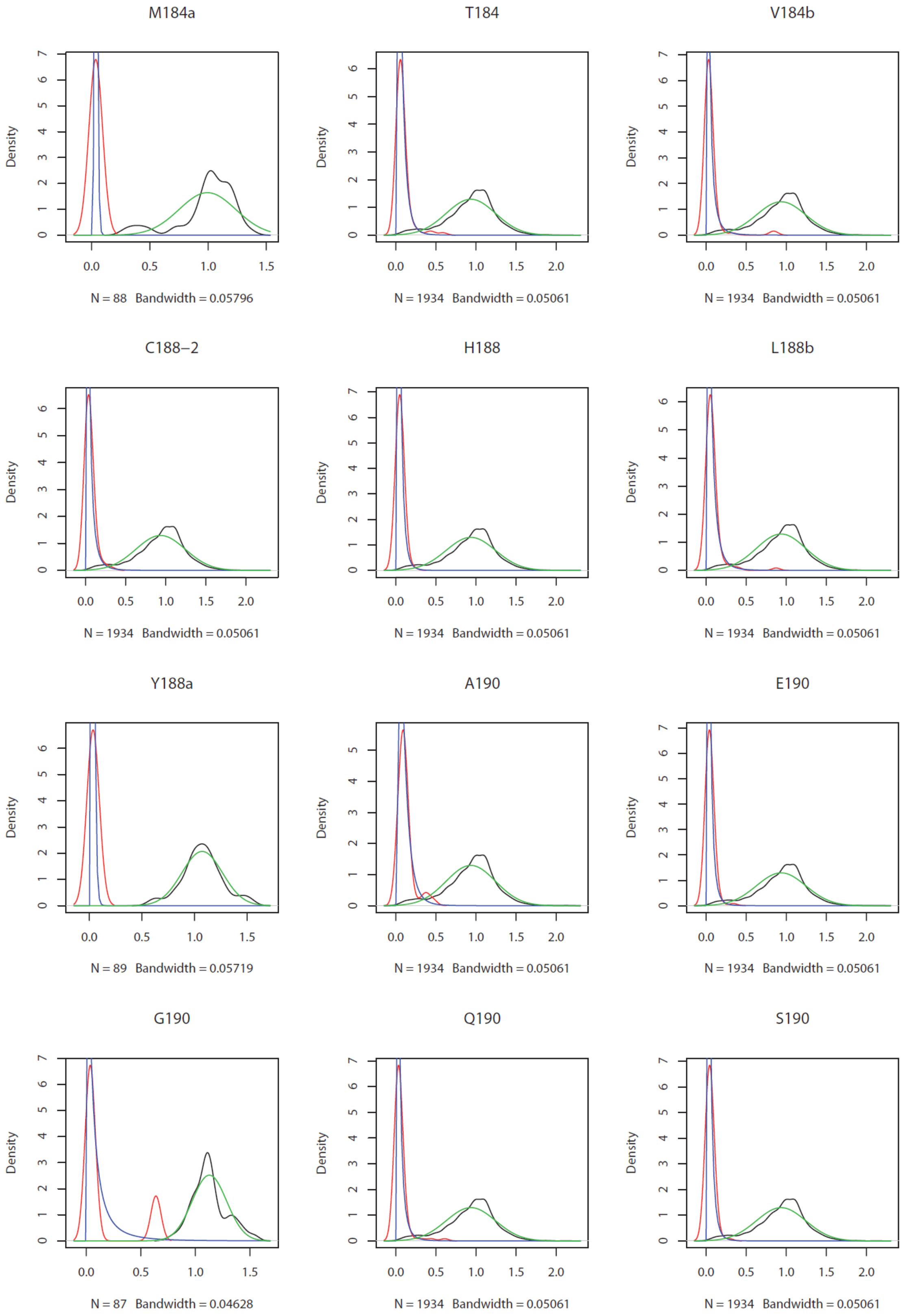
M184a
Density
N = 88 Bandwidth = 0.05796
T184
Density
N = 1934 Bandwidth = 0.05061
V184b
Density
N = 1934 Bandwidth = 0.05061
C188–2
Density
N = 1934 Bandwidth = 0.05061
H188
Density
N = 1934 Bandwidth = 0.05061
L188b
Density
N = 1934 Bandwidth = 0.05061
Y188a
Density
N = 89 Bandwidth = 0.05719
A190
Density
N = 1934 Bandwidth = 0.05061
E190
Density
N = 1934 Bandwidth = 0.05061
G190
Density
N = 87 Bandwidth = 0.04628
Q190
Density
N = 1934 Bandwidth = 0.05061
S190
Density
N = 1934 Bandwidth = 0.05061

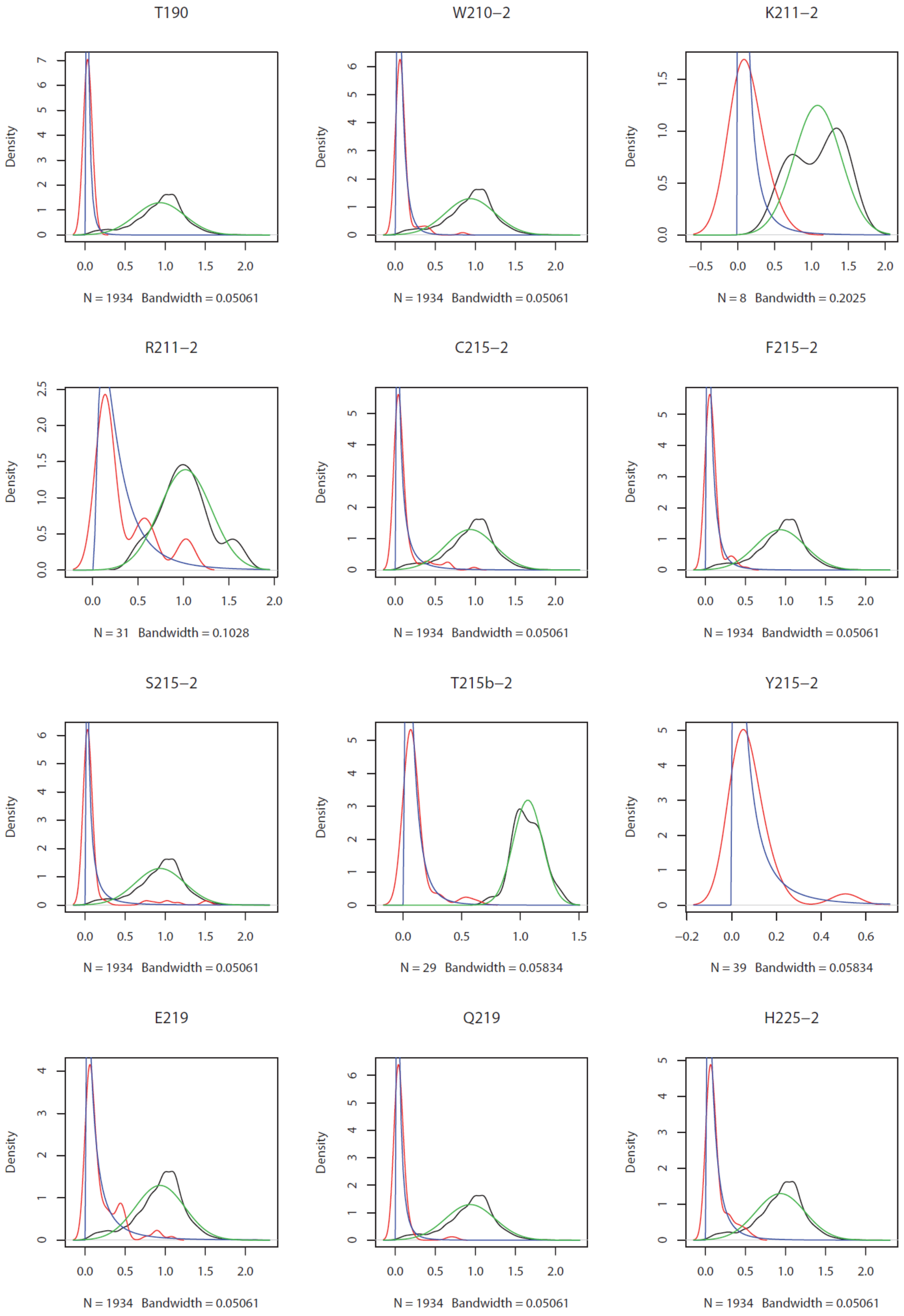

T190
Density
N = 1934 Bandwidth = 0.05061
W210–2
Density
N = 1934 Bandwidth = 0.05061
K211–2
Density
N = 8 Bandwidth = 0.2025
R211–2
Density
N = 31 Bandwidth = 0.1028
C215–2
Density
N = 1934 Bandwidth = 0.05061
F215–2
Density
N = 1934 Bandwidth = 0.05061
S215–2
Density
N = 1934 Bandwidth = 0.05061
T215b–2
Density
N = 29 Bandwidth = 0.05834
Y215–2
Density
N = 39 Bandwidth = 0.05834
E219
Density
N = 1934 Bandwidth = 0.05061
Q219
Density
N = 1934 Bandwidth = 0.05061
H225–2
Density
N = 1934 Bandwidth = 0.05061

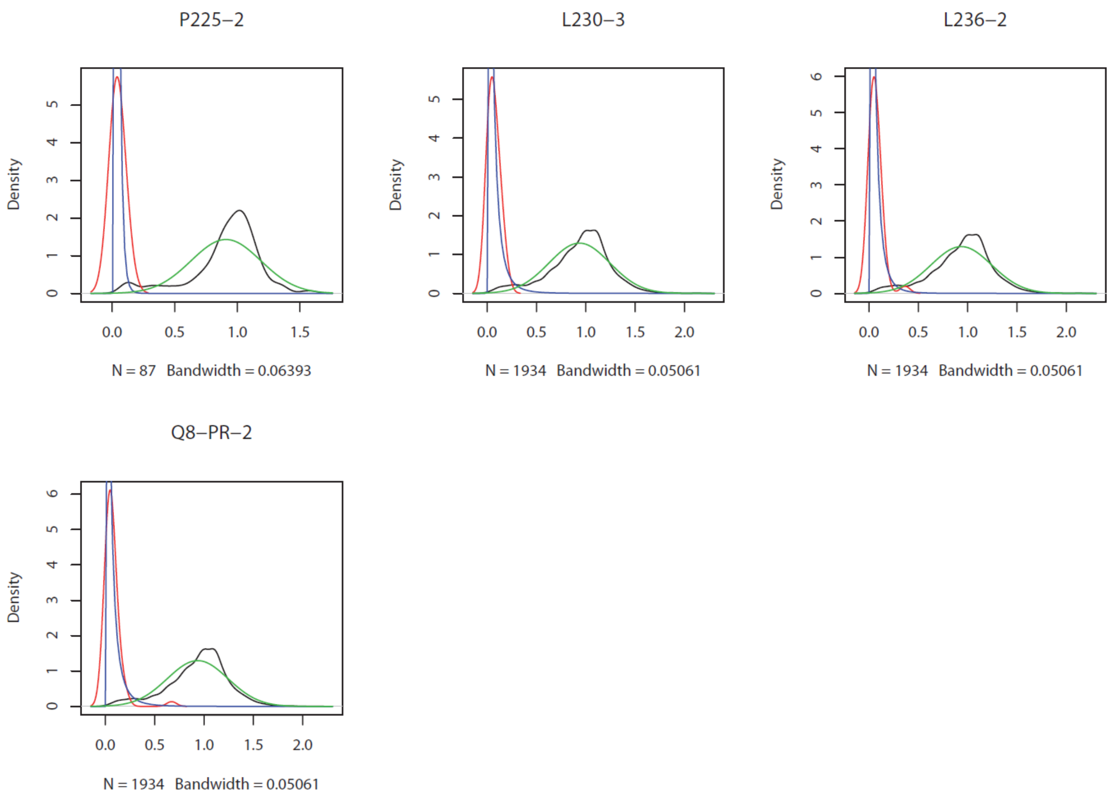
P225−2
Density
N = 87 Bandwidth = 0.06393
L230−3
Density
N = 1934 Bandwidth = 0.05061
L236−2
Density
N = 1934 Bandwidth = 0.05061
Q8−PR−2
Density
N = 1934 Bandwidth = 0.05061

**Figure H. Classification accuracy for the pure clonal samples belonging to the training set.** Columns, probes included in the final version of the microarray that passed the quality control; Rows, samples hybridized to successive versions of the microarray. a) Hybridization of the PR region of the samples; b) Hybridization of the RT region; c) Hybridization of amplicons including both the PR and RT regions. Color code: Dark green, correctly classified signal (TP or TN); Dark red, FN signal; Red, FP signal; Black, UD signal; Light green, no data (due to spots discarded during the quality control or to the absence of certain probes in different versions of the microarray).

a)

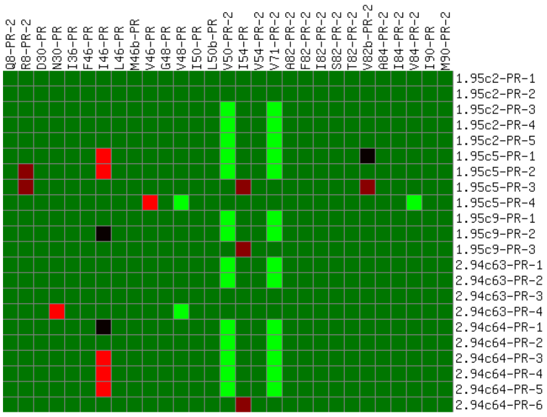

b)

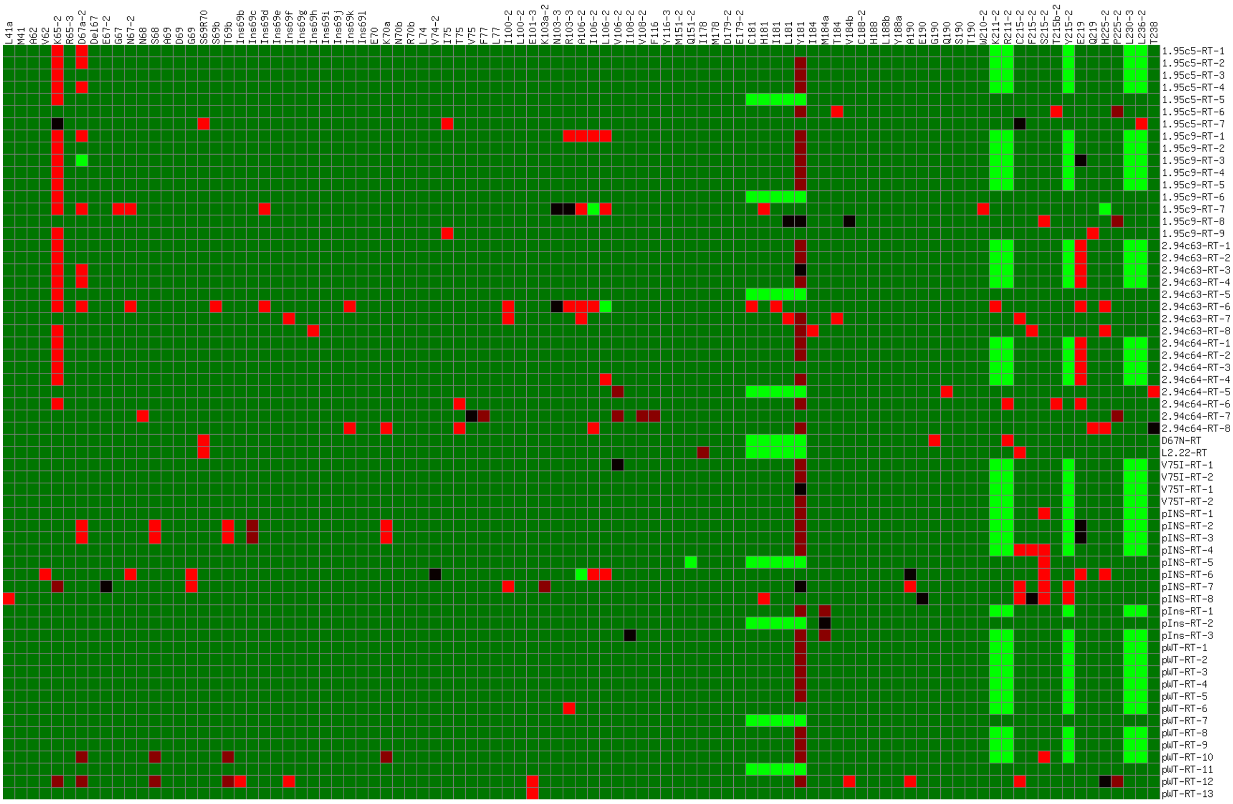

c)

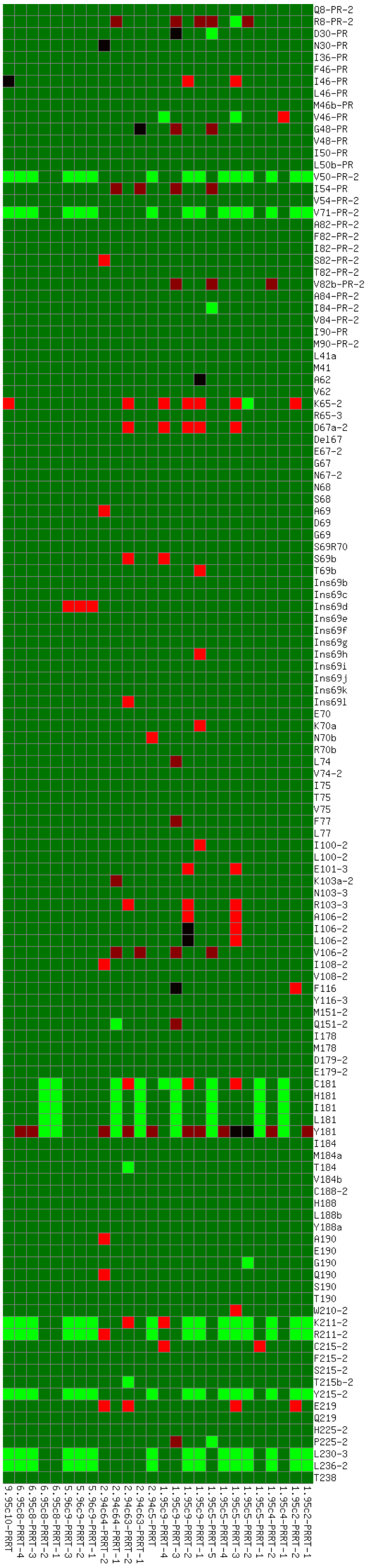
Q8-PR-2
R8-PR-2
D30-PR
N30-PR
I36-PR
F46-PR
I46-PR
L46-PR
M46b-PR
V46-PR
G48-PR
V48-PR
I50-PR
L50b-PR
V50-PR-2
I54-PR
V54-PR-2
V71-PR-2
A82-PR-2
F82-PR-2
I82-PR-2
S82-PR-2
T82-PR-2
V82b-PR-2
A84-PR-2
I84-PR-2
V84-PR-2
I90-PR
M90-PR-2
L41a
M41
A62
V62
K65-2
R65-3
D67a-2
Del67
E67-2
G67
N67-2
N68
S68
A69
D69
G69
S69R70
S69b
T69b
Ins69b
Ins69c
Ins69d
Ins69e
Ins69f
Ins69g
Ins69h
Ins69i
Ins69j
Ins69k
Ins69l
E70
K70a
N70b
R70b
L74
V74-2
I75
T75
V75
F77
L77
I100-2
L100-2
E101-3
K103a-2
N103-3
R103-3
A106-2
I106-2
L106-2
V106-2
I108-2
V108-2
F116
Y116-3
M151-2
Q151-2
I178
M178
D179-2
E179-2
C181
H181
I181
L181
Y181
I184
M184a
T184
V184b
C188-2
H188
L188b
Y188a
A190
E190
G190
Q190
S190
T190
W210-2
K211-2
R211-2
C215-2
F215-2
S215-2
T215b-2
Y215-2
E219
Q219
H225-2
P225-2
L230-3
L236-2
T238
9.95c10-PRRT
6.95c8-PRRT-4
6.95c8-PRRT-3
6.95c8-PRRT-2
6.95c8-PRRT-1
5.96c9-PRRT-3
5.96c9-PRRT-2
5.96c9-PRRT-1
2.94c64-PRRT-2
2.94c64-PRRT-1
2.94c63-PRRT-2
2.94c63-PRRT-1
2.94c5-PRRT
1.95c9-PRRT-4
1.95c9-PRRT-3
1.95c9-PRRT-2
1.95c9-PRRT-1
1.95c5-PRRT-5
1.95c5-PRRT-4
1.95c5-PRRT-3
1.95c5-PRRT-2
1.95c5-PRRT-1
1.95c4-PRRT-2
1.95c4-PRRT-1
1.95c2-PRRT-2
1.95c2-PRRT-1

**Figure I. Accumulation of errors (FP+FN+UD) during classification of clinical samples.** a) Errors per probe (bar: 11 to 20 errors); b) Errors per sample (bar: 10 to 22 errors). The remaining probes and hybridized samples showed a lower number of errors.

a)

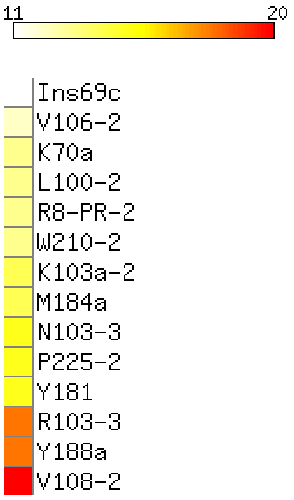


b)

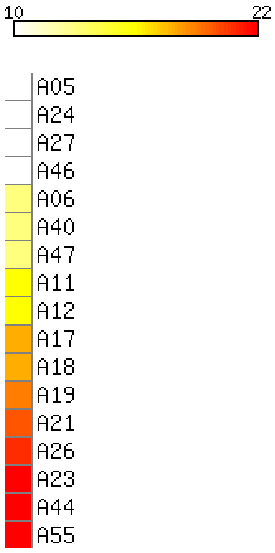

**Figure J. Theoretical hybridization tables of binary mixtures of clonal samples belonging to the training set.** a) Samples 1.95c9, 2.94c64 and their mixture; b) Samples pWT, pINS and their mixture. Color code: Green, expected hybridization; Red, not expected hybridization; Black, partial hybridization (one mismatch between probe and target is present at the 5’ or 3’ nucleotide of the hybridizing sequence).

a)

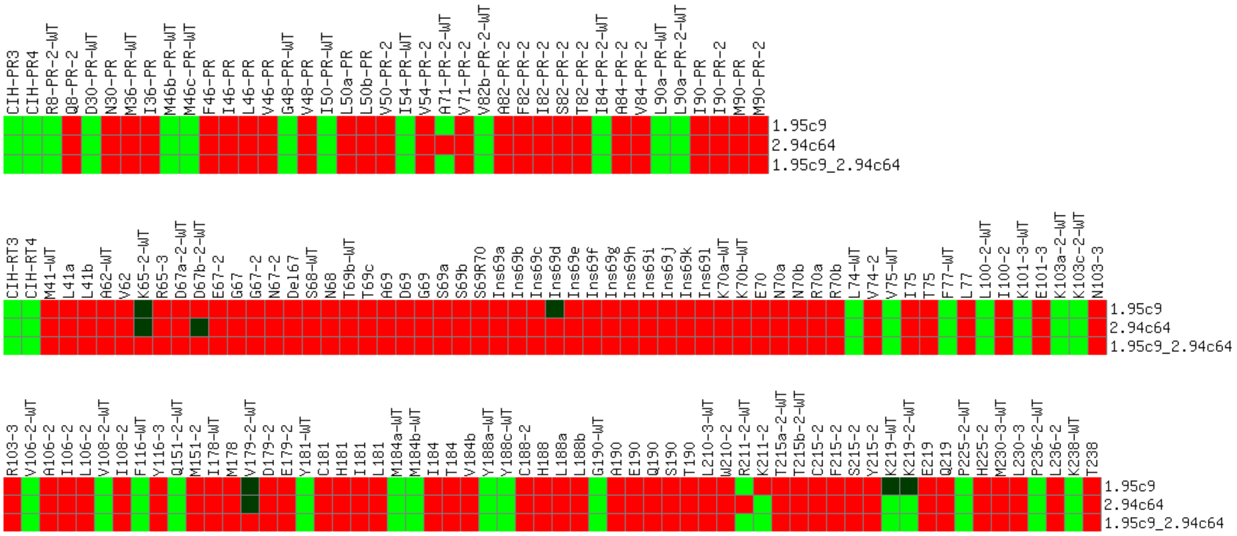


b)

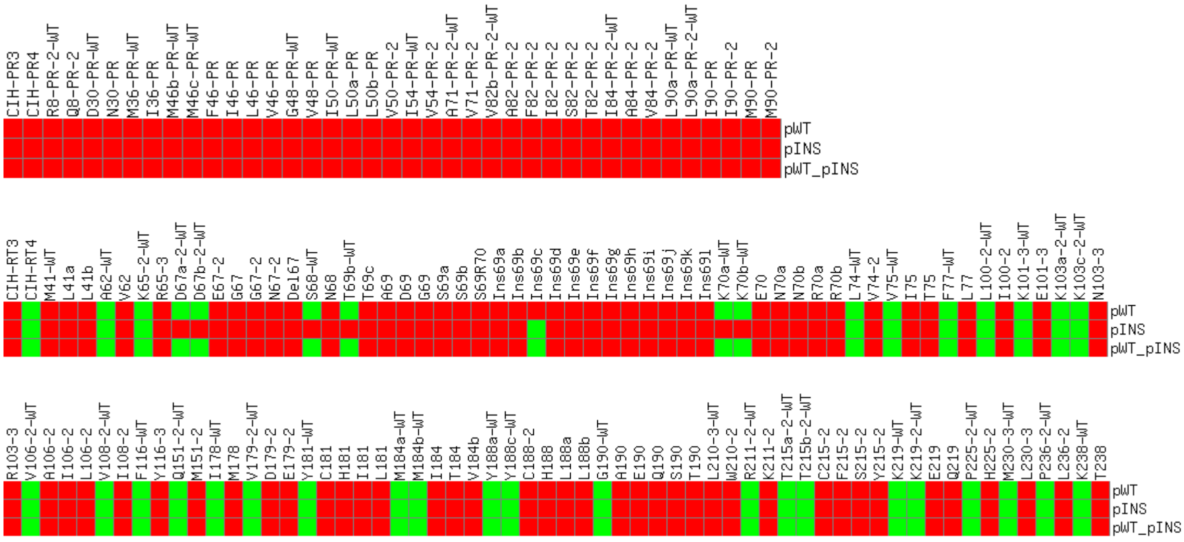

**Figure K. Proportion of variants present in the clinical samples of the test set, determined by clonal sequencing.** a) Probes of the PR region (columns) vs. sequences of the 53 target molecules that could be amplified out of the 57 clinical samples (rows). b) Probes of the RT region vs. sequences of the 51 amplified RT clinical samples. Bar: fraction of codons containing each queried codon within the quasispecies. c) Distribution of variant proportions among the target samples.

a)

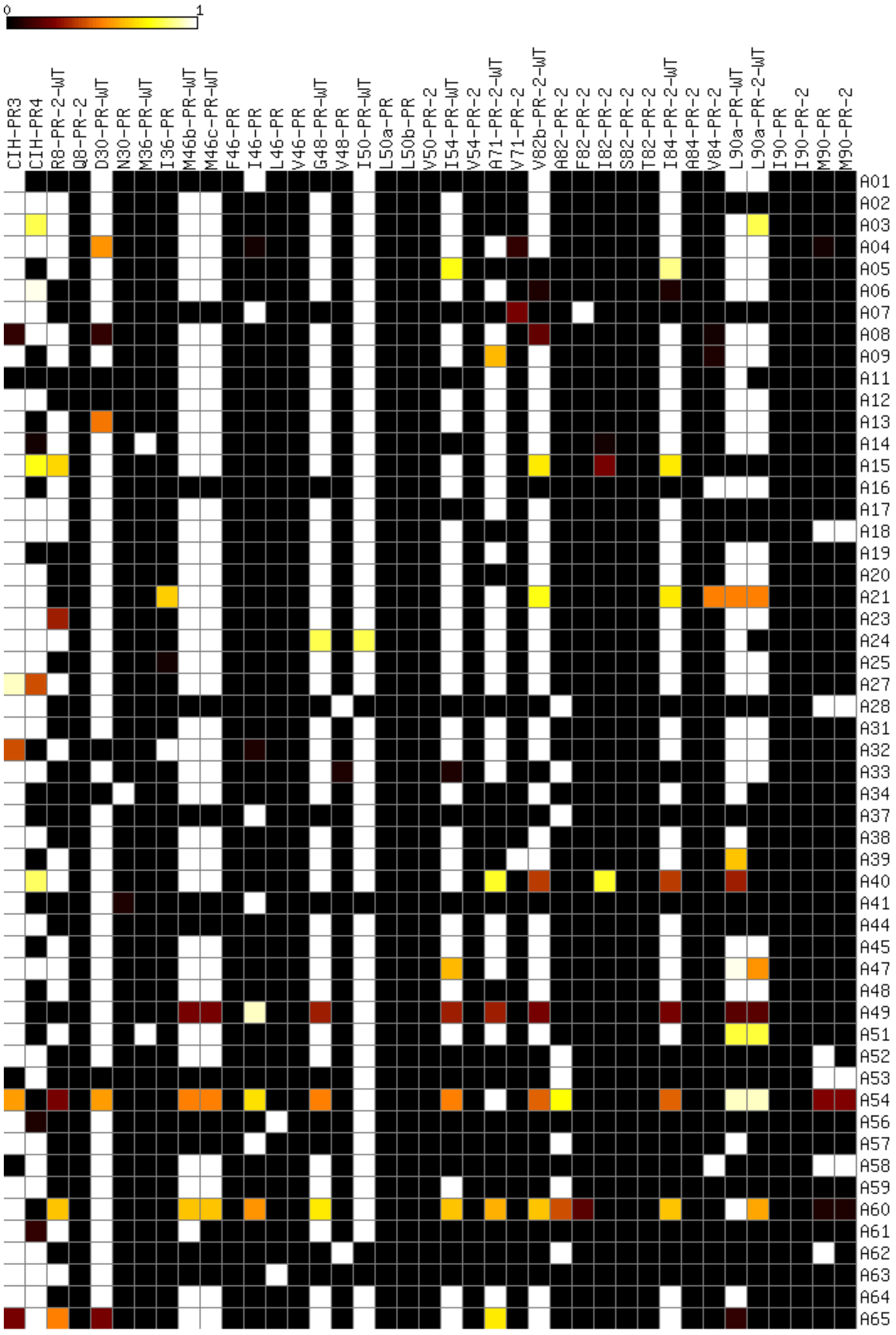

b)

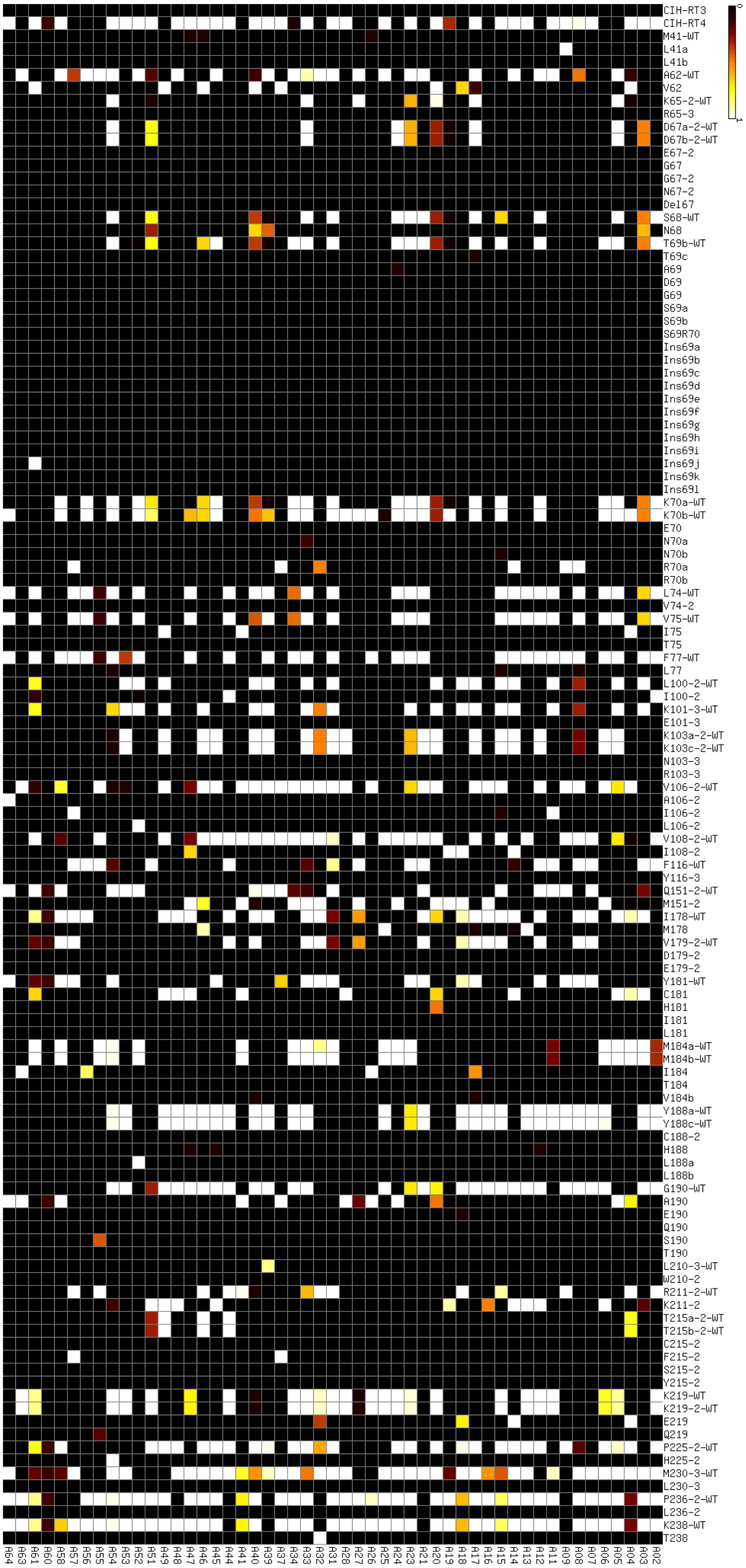
CIH-RT3
CIH-RT4
M41-WT
L41a
L41b
A62-WT
V62
K65-2-WT
R65-3
D67a-2-WT
D67b-2-WT
E67-2
G67
G67-2
N67-2
Del67
S68-WT
N68
T69b-WT
T69c
A69
D69
G69
S69a
S69b
S69R70
Ins69a
Ins69b
Ins69c
Ins69d
Ins69e
Ins69f
Ins69g
Ins69h
Ins69i
Ins69j
Ins69k
Ins69l
K70a-WT
K70b-WT
E70
N70a
N70b
R70a
R70b
L74-WT
V74-2
V75-WT
I75
T75
F77-WT
L77
L100-2-WT
I100-2
K101-3-WT
E101-3
K103a-2-WT
K103c-2-WT
N103-3
R103-3
V106-2-WT
A106-2
I106-2
L106-2
V108-2-WT
I108-2
F116-WT
Y116-3
Q151-2-WT
M151-2
I178-WT
M178
V179-2-WT
D179-2
E179-2
Y181-WT
C181
H181
I181
L181
M184a-WT
M184b-WT
I184
T184
V184b
Y188a-WT
Y188c-WT
C188-2
H188
L188a
L188b
G190-WT
A190
E190
Q190
S190
T190
L210-3-WT
W210-2
R211-2-WT
K211-2
T215a-2-WT
T215b-2-WT
C215-2
F215-2
S215-2
Y215-2
K219-WT
K219-2-WT
E219
Q219
P225-2-WT
H225-2
M230-3-WT
L230-3
P236-2-WT
L236-2
K238-WT
T238
A64 A63 A61 A60 A58 A57 A56 A55 A54 A53 A52 A51 A49 A48 A47 A46 A45 A44 A41 A40 A39 A37 A34 A33 A32 A31 A28 A27 A26 A25 A24 A23 A21 A20 A19 A18 A17 A16 A15 A14 A13 A12 A11 A09 A08 A07 A06 A05 A04 A03 A02
0
1

c)

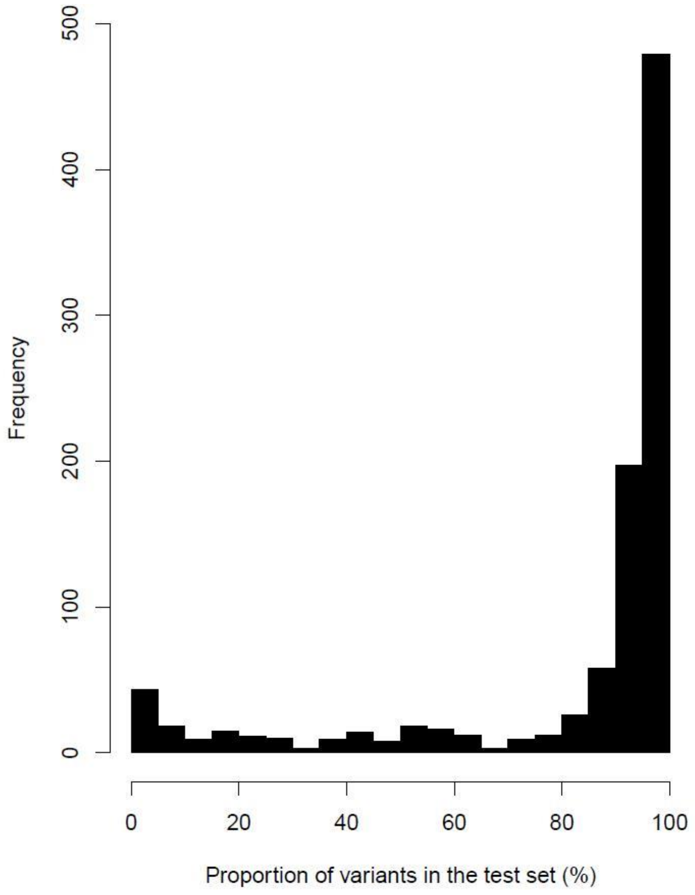

Frequency
500
400
300
200
100
0
0
20
40
60
80
100
Proportion of variants in the test set (%)

**Figure L. Theoretical hybridization tables of the test set, using a preliminary detection threshold of 10% for minority sequences.** Only perfect probe-target hybridizations are allowed. Columns, queried codons included in probes belonging to the PR (a) or RT (b) region; Rows, sequence of the quasispecies of each clinical sample (see legend of Supp. Fig. S11). Color code: Green, expected hybridization (>10% of the clonal sequences of the sample match with the probe); Black, residual hybridization (probe matching in 5-10% of the sample clones); Red, not expected hybridization.

a)

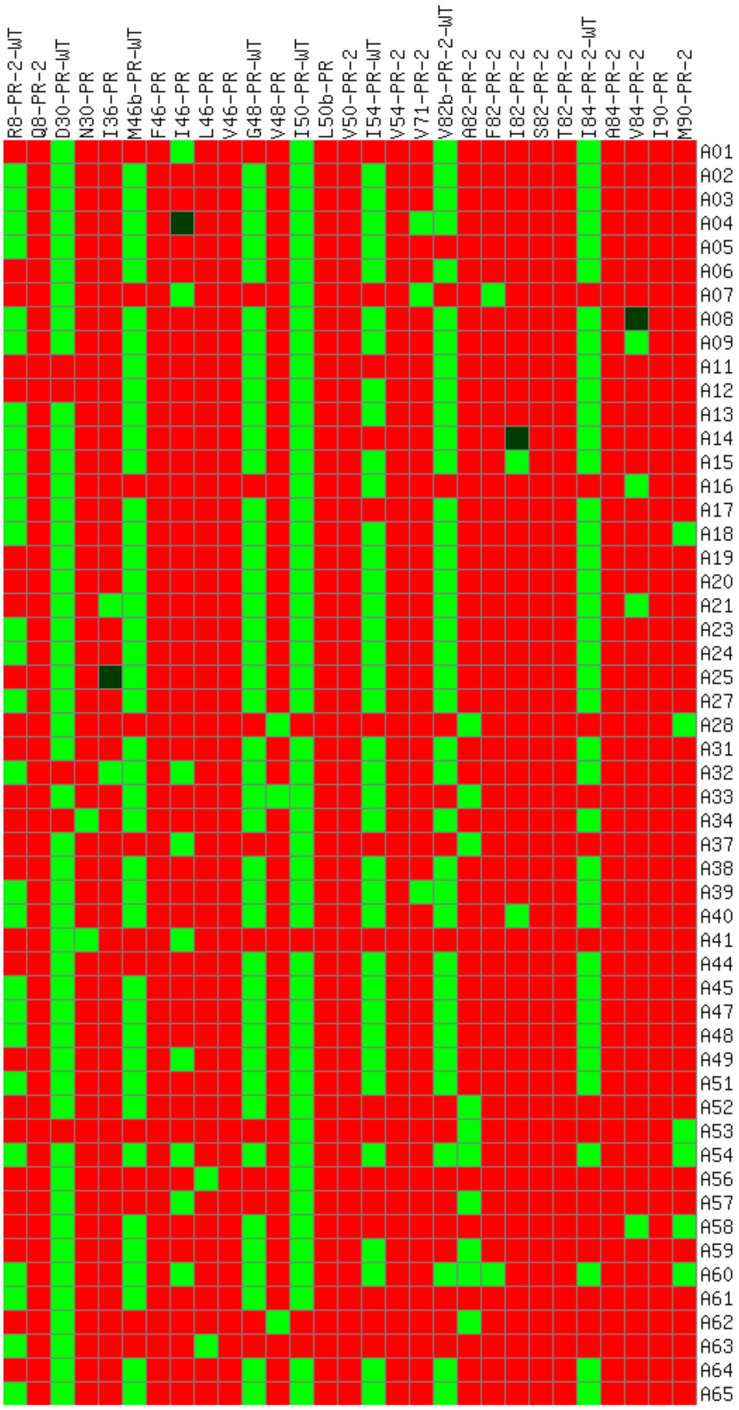

b)

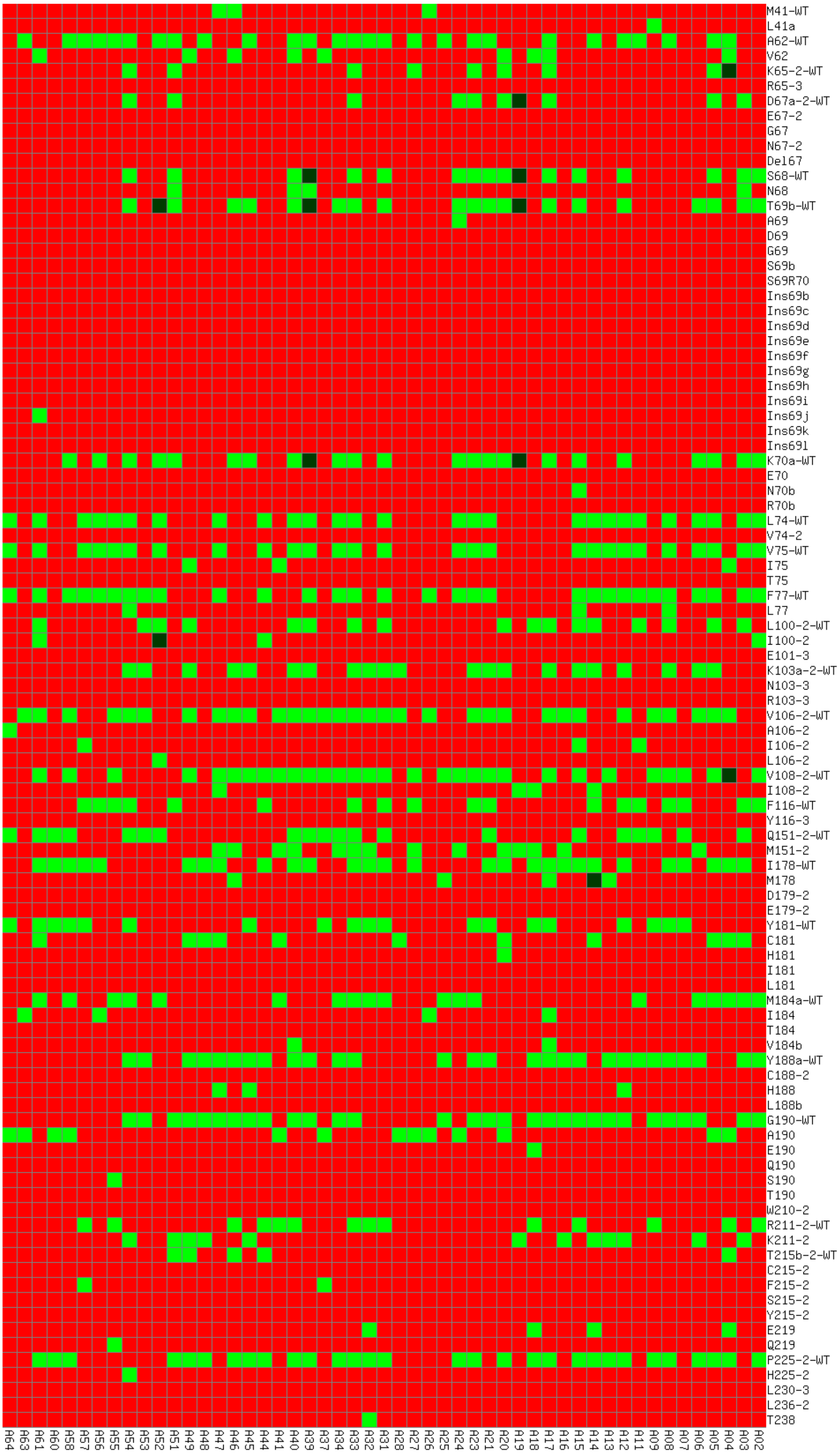
M41-WT
L41a
A62-WT
V62
K65-2-WT
R65-3
D67a-2-WT
E67-2
G67
N67-2
Del67
S68-WT
N68
T69b-WT
A69
D69
G69
S69b
S69R70
Ins69b
Ins69c
Ins69d
Ins69e
Ins69f
Ins69g
Ins69h
Ins69i
Ins69j
Ins69k
Ins69l
K70a-WT
E70
N70b
R70b
L74-WT
V74-2
V75-WT
I75
T75
F77-WT
L77
L100-2-WT
I100-2
E101-3
K103a-2-WT
N103-3
R103-3
V106-2-WT
A106-2
I106-2
L106-2
V108-2-WT
I108-2
F116-WT
Y116-3
Q151-2-WT
M151-2
I178-WT
M178
D179-2
E179-2
Y181-WT
C181
H181
I181
L181
M184a-WT
I184
T184
V184b
Y188a-WT
C188-2
H188
L188b
G190-WT
A190
E190
Q190
S190
T190
W210-2
R211-2-WT
K211-2
T215b-2-WT
C215-2
F215-2
S215-2
Y215-2
E219
Q219
P225-2-WT
H225-2
L230-3
L236-2
T238
A64
A63
A61
A60
A58
A57
A56
A55
A54
A53
A52
A51
A49
A48
A47
A46
A45
A44
A41
A40
A39
A37
A34
A33
A32
A31
A28
A27
A26
A25
A24
A23
A21
A20
A19
A18
A17
A16
A15
A14
A13
A12
A11
A09
A08
A07
A06
A05
A04
A03
A02

**Table A. Sequences of the oligonucleotide probes printed onto the microarray for the screening of HIV-1 PR and RT drug and multidrug-resistance mutations.** Discriminating probes are shown in black, positive HIV-1 internal hybridization controls (IHC-PR3, IHC-PR4, IHC-RT3 and IHC-RT4, spanning codons 25-29 and 94-99 of the PR, as well as 23-28 and 167-171 of the RT, respectively) are depicted in green, and negative controls (FMDV-G142-15r and FMDV-E142-15r, corresponding to sequences of the foot-and-mouth disease virus VP1 coding region) are shown in red. Yellow background highlights the probes complementary to wild type sequences at each queried codon. Two probes containing degenerated nucleotides (Ins69k and Ins69l) that could hybridize with a number of different targets showing 6 nt insertions between RT codons 69 and 70 have been included. The 124 highly performing probes that were maintained after the quality control protocol (see main text and Supp. Fig. S7) are underlined. In the second column, the nucleotide mutation(s) with respect to the corresponding wild type sequence are marked in bold. The calculated, salt-adjusted melting temperatures (quantified by means of the software available at http://www.basic.northwestern.edu/biotools/oligocalc.html) are shown.

| Oligo name | Nucleotide sequence (5'-3') | Tm (ºC) |
|---|---|---|
| IHC-PR3 | TTTTTTTTTTTTTTTGATACAGGAGCAGAT | 54 |
| IHC-PR4 | TTTTTTTTTTTTTTTGTTGCACTTTAAATTTT | 51 |
| R8-PR-2 | TTTTTTTTTTTTTTTGCAACGACCCCTC | 55 |
| Q8-PR-2 | TTTTTTTTTTTTTTTGCAAC**A**ACCCCTC | 53 |
| D30-PR | TTTTTTTTTTTTTTTAGCAGATGATACAGT | 51 |
| N30-PR | TTTTTTTTTTTTTTTAGCAGAT**A**ATACAGT | 49 |
| M36-PR | TTTTTTTTTTTTTTTAAGAAATGAGTTTGC | 50 |
| I36-PR | TTTTTTTTTTTTTTTAAGAAAT**A**AGTTTGC | 49 |
| M46b-PR | TTTTTTTTTTTTTTTCAAAAATGATAGGGG | 51 |
| M46c-PR | TTTTTTTTTTTTTTTACCAAAAATGATAGG | 49 |
| F46-PR | TTTTTTTTTTTTTTTCCAAAA**T**TCATAGGG | 51 |
| I46-PR | TTTTTTTTTTTTTTTCAAAAAT**A**ATAGGGG | 49 |
| L46-PR | TTTTTTTTTTTTTTTACCAAAA**T**TGATAGG | 49 |
| V46-PR | TTTTTTTTTTTTTTTACCAAAA**G**TGATAGG | 51 |
| G48-PR | TTTTTTTTTTTTTTTATGATAGGGGGAATT | 51 |
| V48-PR | TTTTTTTTTTTTTTTATGATAG**T**GGGAATT | 49 |
| I50-PR | TTTTTTTTTTTTTTTGGGGAATTGGAG | 49 |
| L50a-PR | TTTTTTTTTTTTTTTGGGGA**T**TAGGAG | 49 |
| L50b-PR | TTTTTTTTTTTTTTTGGGA**C**TCGGAG | 49 |
| V50-PR-2 | TTTTTTTTTTTTTTTGGTGA**G**TTGGAG | 49 |
| I54-PR | TTTTTTTTTTTTTTTAGGTTTTATCAAAGTA | 49 |
| V54-PR-2 | TTTTTTTTTTTTTTTAGGTATT**G**TCAAAGTA | 51 |
| A71-PR-2 | TCCTCCTCCTCCTCCCATAAAGCTATAGGTA | 51 |
| V71-PR-2 | TCCTCCTCCTCCTCCCATAAAG**T**TATAGGTA | 49 |
| V82b-PR-2 | TCCTCCTCCTCCTCCTACACCTGTCAACAT | 51 |
| A82-PR-2 | TCCTCCTCCTCCTCCACACCTG**C**CAACAT | 52 |
| F82-PR-2 | TCCTCCTCCTCCTCCTACACC**T**TTCAACAT | 49 |

| | | |
|---|---|---|
| I82-PR-2 | TCCTCCTCCTCCTCCTACACCT**A**TCAACAT | 49 |
| S82-PR-2 | TCCTCCTCCTCCTCCTACACCT**TC**CAACAT | 51 |
| T82-PR-2 | TCCTCCTCCTCCTCCTACACCT**AC**CAACAT | 51 |
| I84-PR-2 | TCCTCCTCCTCCTCCTGTCAACATAATTGG | 49 |
| A84-PR-2 | TCCTCCTCCTCCTCCGTCAAC**GC**AATTGG | 52 |
| V84-PR-2 | TCCTCCTCCTCCTCCGTCAAC**G**TAATTGG | 49 |
| L90a-PR | TTTTTTTTTTTTTTTAATCTGTTGACTCAG | 51 |
| L90a-PR-2 | TTTTTTTTTTTTTTTAATCTGTTGACTCAGA | 54 |
| I90-PR | TTTTTTTTTTTTTTTAATCTG**A**T**A**ACTCAG | 49 |
| I90-PR-2 | TTTTTTTTTTTTTTTAATCTG**A**T**A**ACTCAGA | 51 |
| M90-PR | TTTTTTTTTTTTTTTAAATCTG**A**TGACTCA | 49 |
| M90-PR-2 | TTTTTTTTTTTTTTTAATCTG**A**TGACTCAGA | 54 |
| IHC-RT3 | TTTTTTTTTTTTTTTATGGCCATTGACAGA | 54 |
| IHC-RT4 | TTTTTTTTTTTTTTTATCTTAGAGCCTTTTA | 51 |
| M41 | TTTTTTTTTTTTTTTTACAGAGATGGAAAA | 49 |
| L41a | TTTTTTTTTTTTTTTTACAGAG**T**TGGAAAA | 49 |
| L41b | TTTTTTTTTTTTTTTTACAGAG**C**TGGAAAA | 51 |
| A62 | TTTTTTTTTTTTTTTAGTATTTGCCATAAAG | 51 |
| V62 | TTTTTTTTTTTTTTTAGTATTTG**T**CATAAAG | 49 |
| K65-2 | TCCTCCTCCTCCTCCCATAAAGAAAAAAGAC | 49 |
| R65-3 | TCCTCCTCCTCCTCCCATAAAGA**G**AATAGAC | 51 |
| D67a-2 | TCCTCCTCCTCCTCCAAAAAAGACAGTACTA | 49 |
| D67b-2 | TCCTCCTCCTCCTCCGAAAAAAGACAGTACT | 51 |
| E67-2 | TCCTCCTCCTCCTCCAAAAAAGA**A**AGTACTAA | 49 |
| G67 | TTTTTTTTTTTTTTTAAAAAAG**G**CAGTACTA | 51 |
| G67-2 | TCCTCCTCCTCCTCCAAAAAAG**G**CAGTACTA | 51 |
| N67-2 | TCCTCCTCCTCCTCCGAAAAAA**A**ACAGTACT | 49 |
| Del67 | TTTTTTTTTTTTTTTAAGAAAA**A**A**A**GTACTAA | 49 |
| S68 | TTTTTTTTTTTTTTTAAAAGACAGTACTAAAT | 51 |
| N68 | TTTTTTTTTTTTTTTAAAAGACA**A**TACTAAAT | 49 |
| T69b | TTTTTTTTTTTTTTTGACAGTACTAAATGG | 51 |
| T69c | TTTTTTTTTTTTTTTGACAGTAC**A**AAATGG | 51 |
| A69 | TTTTTTTTTTTTTTTAGACAGT**G**CTAAATG | 51 |
| D69 | TTTTTTTTTTTTTTTGACAGT**GA**TAAATGG | 51 |
| G69 | TTTTTTTTTTTTTTTGACAGT**GG**TAAATG | 49 |
| S69a | TTTTTTTTTTTTTTTGACAGTA**G**TAAATGG | 51 |
| S69b | TTTTTTTTTTTTTTTGACAGTA**GA**AAATGG | 51 |
| S69R70 | TTTTTTTTTTTTTTTACAGT**T**CTAGATGGA | 51 |
| Ins69a | TTTTTTTTTTTTTTTA**G**T**AGTAGT**AGTAAAT | 49 |
| Ins69b | TTTTTTTTTTTTTTTGT**T**CT**AGTTCT**AGAT | 49 |
| Ins69c | TTTTTTTTTTTTTTTT**CG**A**G**T**AGTTCG**AAA | 51 |
| Ins69d | TTTTTTTTTTTTTTTAGTA**G**T**AGTGGT**AAA | 49 |
| Ins69e | TTTTTTTTTTTTTTTTAGT**GG**T**AGATGG** | 49 |
| Ins69f | TTTTTTTTTTTTTTTAGTA**G**T**AGCGCT**AAA | 51 |
| Ins69g | TTTTTTTTTTTTTTTACT**AGCAGCGCTA** | 51 |
| Ins69h | TTTTTTTTTTTTTTTAGTA**G**T**GAAGCA**AAA | 49 |
| Ins69i | TTTTTTTTTTTTTTTAGT**T**CT**ACCTCT**A**G**A | 51 |

| | | |
|---|---|---|
| Ins69j | TTTTTTTTTTTTTTTAGTA**GCGTGACT**AAA | 51 |
| Ins69k | TTTTTTTTTTTTTTTAGTACT**AGTDST**AAAT | 49-51 |
| Ins69l | TTTTTTTTTTTTTTTGT**WS**T**AGTDST**A**R**AT | 46-51 |
| K70a | TTTTTTTTTTTTTTTCAGTACTAAATGGAG | 51 |
| K70b | TTTTTTTTTTTTTTTGTACTAAATGGAGAA | 49 |
| E70 | TTTTTTTTTTTTTTTCAGTACT**G**AATGGA | 49 |
| N70a | TTTTTTTTTTTTTTTGTACTAA**T**TGGAGAA | 49 |
| N70b | TTTTTTTTTTTTTTTGTACTAA**C**TGGAGAA | 51 |
| R70a | TTTTTTTTTTTTTTTAGTACTA**G**ATGGAGA | 51 |
| R70b | TTTTTTTTTTTTTTTGTACTA**GG**TGGAG | 51 |
| L74 | TTTTTTTTTTTTTTTGAGAAAATTAGTAGAT | 49 |
| V74-2 | TTTTTTTTTTTTTTTGAGAATA**G**TAGTAGAT | 51 |
| V75 | TTTTTTTTTTTTTTTGAAAATTAGTAGATTTC | 51 |
| I75 | TTTTTTTTTTTTTTTGAAAATTA**A**TAGATTTC | 49 |
| T75 | TTTTTTTTTTTTTTTGAAAATTA**AC**AGATTTC | 51 |
| F77 | TTTTTTTTTTTTTTTAGTAGATTTCAGAGA | 49 |
| L77 | TTTTTTTTTTTTTTTAGTAGAT**C**TCAGAGA | 51 |
| L100-2 | TCCTCCTCCTCCTCCCGCAGGGTTAAAAAA | 51 |
| I100-2 | TCCTCCTCCTCCTCCCGCAGGG**A**TAAAAAA | 51 |
| K101-3 | TCCTCCTCCTCCTCCGCAGGGTTAAAAAAGA | 54 |
| E101-3 | TCCTCCTCCTCCTCCGCAGGTTTA**G**AAAAGA | 54 |
| K103a-2 | TCCTCCTCCTCCTCCAAAAAGAAAAAATCAGT | 49 |
| K103c-2 | TCCTCCTCCTCCTCCAAAAAAGAAAAAATCAG | 49 |
| N103-3 | TCCTCCTCCTCCTCCAAATAGAA**C**AAATCAGT | 51 |
| R103-3 | TCCTCCTCCTCCTCCAAAATAGA**G**AAAATCAG | 51 |
| V106-2 | TCCTCCTCCTCCTCCAAAATCAGTAACAGTA | 49 |
| A106-2 | TCCTCCTCCTCCTCCAAAATCAG**C**AACAGTA | 51 |
| I106-2 | TCCTCCTCCTCCTCCAAAAATCA**A**TAACAGTA | 49 |
| L106-2 | TCCTCCTCCTCCTCCAAAAATCA**T**TAACAGTA | 49 |
| V108-2 | TTTTTTTTTTTTTTTCAGTAACAGTACTGG | 54 |
| I108-2 | TTTTTTTTTTTTTTTCAGTAACA**A**TACTGG | 51 |
| F116 | TTTTTTTTTTTTTTTGCATATTTTTCAGTTC | 51 |
| Y116-3 | TTTTTTTTTTTTTTTGCATATT**A**TTCACTTC | 51 |
| Q151-2 | TTTTTTTTTTTTTTTGCTTCCACAGGGAT | 55 |
| M151-2 | TTTTTTTTTTTTTTTGCTTCCA**AT**GGGAT | 52 |
| I178 | TTTTTTTTTTTTTTTCAGACATAGTTATCT | 49 |
| M178 | TTTTTTTTTTTTTTTCAGACAT**G**GTTATCT | 51 |
| V179-2 | TTTTTTTTTTTTTTTAGACATAGTTATCTATC | 53 |
| D179-2 | TTTTTTTTTTTTTTTAGACATAG**A**TATCTATC | 53 |
| E179-2 | TTTTTTTTTTTTTTTAGACATAG**AG**ATCTAT | 51 |
| Y181 | TTTTTTTTTTTTTTTAGTTATCTATCAATAC | 49 |
| C181 | TTTTTTTTTTTTTTTAGTTATCT**G**TCAATAC | 51 |
| H181 | TTTTTTTTTTTTTTTAGTTATC**C**ATCAATAC | 51 |
| I181 | TTTTTTTTTTTTTTTAGTTATC**AT**TCAATAC | 49 |
| L181 | TTTTTTTTTTTTTTTAGTTATC**CT**TCAATAC | 51 |
| M184a | TTTTTTTTTTTTTTTCAATACATGGATGATT | 51 |
| M184b | TTTTTTTTTTTTTTTCAATACATGGATGAT | 49 |

| | | |
|---|---|---|
| **I184** | TTTTTTTTTTTTTTTCAATACAT**A**GATGATT | 49 |
| **T184** | TTTTTTTTTTTTTTTCAATACA**C**GGATGAT | 51 |
| **V184b** | TTTTTTTTTTTTTTTCAATAC**G**T**A**GATGAT | 49 |
| **Y188a** | TTTTTTTTTTTTTTTGATTTGTATGTAGGA | 49 |
| **Y188c** | TTTTTTTTTTTTTTTGATTTGTATGTAGGAT | 51 |
| **C188-2** | TTTTTTTTTTTTTTTGATTAGT**G**TGTAGGA | 51 |
| **H188** | TTTTTTTTTTTTTTTTGATTTG**C**ATGTAGG | 51 |
| **L188a** | TTTTTTTTTTTTTTTGATTTGT**TA**GTAGGAT | 51 |
| **L188b** | TTTTTTTTTTTTTTTTGATTTG**CT**TGTAGG | 51 |
| **G190** | TTTTTTTTTTTTTTTTATGTAGGATCTGAC | 51 |
| **A190** | TTTTTTTTTTTTTTTTATGTAG**C**ATCTGAC | 51 |
| **E190** | TTTTTTTTTTTTTTTTATGTAG**A**ATCTGAC | 49 |
| **Q190** | TTTTTTTTTTTTTTTTATGTA**CA**ATCTGAC | 49 |
| **S190** | TTTTTTTTTTTTTTTTATGTA**A**G**C**TCTGAC | 51 |
| **T190** | TTTTTTTTTTTTTTTTATGTAACATCTGAC | 49 |
| **L210-3** | TTTTTTTTTTTTTTTATCTGTTGAGTTGGG | 54 |
| **W210-2** | TTTTTTTTTTTTTTTATCTGT**G**GAGTTGG | 52 |
| **R211-2** | TTTTTTTTTTTTTTTGTTGAGGTGGGGA | 53 |
| **K211-2** | TTTTTTTTTTTTTTTGTTGA**A**GTGGGGA | 51 |
| **T215a-2** | TTTTTTTTTTTTTTTGGACTTACCACACC | 55 |
| **T215b-2** | TTTTTTTTTTTTTTTGGGACTTACCACAC | 55 |
| **C215-2** | TTTTTTTTTTTTTTTGGACTT**TG**CACACC | 55 |
| **F215-2** | TTTTTTTTTTTTTTTGGACTT**TT**CACACC | 52 |
| **S215-2** | TTTTTTTTTTTTTTTGGGACTT**T**CCACAC | 55 |
| **Y215-2** | TTTTTTTTTTTTTTTGGACTT**TA**CACACC | 52 |
| **K219** | TTTTTTTTTTTTTTTACCAGACAAAAAACA | 49 |
| **K219-2** | TCCTCCTCCTCCTCCACCAGACAAAAAACA | 49 |
| **E219** | TTTTTTTTTTTTTTTACCAGAC**G**AAAAACA | 51 |
| **Q219** | TTTTTTTTTTTTTTTACCAGAC**C**AAAAACA | 51 |
| **P225-2** | TCCTCCTCCTCCTCCAAAGAACCTCCATTC | 51 |
| **H225-2** | TCCTCCTCCTCCTCCAAAGAAC**A**TCCATTC | 49 |
| **M230-3** | TTTTTTTTTTTTTTTCTTTGGATGGGTTAT | 51 |
| **L230-3** | TTTTTTTTTTTTTTTCTTTGG**C**TGGGTTAT | 54 |
| **P236-2** | TCCTCCTCCTCCTCCCTCCATCCTGATAAAT | 54 |
| **L236-2** | TCCTCCTCCTCCTCCCTCCATC**T**TGATAAAT | 51 |
| **K238** | TTTTTTTTTTTTTTTCCTGATAAATGGAC | 49 |
| **T238** | TTTTTTTTTTTTTTTCCTGATA**C**ATGGAC | 51 |
| **FMDV-G142-15r** | TTTTTTTTTTTTTTTCAAATCCCCGCGTG | 56 |
| **FMDV-E142-15r** | TTTTTTTTTTTTTTTCAAATCCTCGCGTG | 54 |

**Table B. Classification accuracy of binary mixtures of pure clonal samples used to set the preliminary detection limit of the microarray.** Step 4 did not increase the accuracy in this series of experiments because none of the full hybridized microarrays had to be discarded.

| **Signals** | **Classification accuracy (% of signals) after filtering** | | | | | | | |
|---|---|---|---|---|---|---|---|---|
| | **Step 1: Spot filter** | | **Step 2: Probe filter 1 (overlapped)** | | **Step 3: Probe filter 2 (duplications)** | | **Step 4: Array filter** | |
| | 1.95c9/ 2.94c64 (RT and PR-RT) | pWT/ pINS (RT) | 1.95c9/ 2.94c64 (RT and PR-RT) | pWT/ pINS (RT) | 1.95c9/ 2.94c64 (RT and PR-RT) | pWT/ pINS (RT) | 1.95c9/ 2.94c64 (RT and PR-RT) | pWT/ pINS (RT) |
| Correct | 95.32 | 91.15 | 97.80 | 93.08 | 97.64 | 94.26 | 97.64 | 94.26 |
| FP | 2.61 | 3.35 | 1.37 | 2.13 | 1.50 | 1.44 | 1.50 | 1.44 |
| FN | 1.44 | 4.77 | 0.49 | 3.93 | 0.54 | 3.36 | 0.54 | 3.36 |
| UD | 0.62 | 0.73 | 0.34 | 0.86 | 0.32 | 0.93 | 0.32 | 0.93 |

**Table C. Minority subpopulations present in the clinical samples of the test set, quantified by means of clonal analysis.**

| **Range of minority variants within the quasispecies (%)** | **Fraction of positions (PR + RT)** |
|---|---|
| 1.00 – 4.99 | 15/970 (1.55%) |
| 5.00 – 9.99 | 34/970 (3.50%) |
| 10.00 – 19.99 | 24/970 (2.47%) |

**Table D. Codons of the HIV-1 PR (a) and RT (b) regions of the *pol* gene queried by the genotyping microarray (detailed in Supp. Table S1) compared to the current update of the drug resistance mutations to PIs, NRTIs and NNRTIs (Wensing et al., 2015; mutations conferring low level resistance have been stripped).** Abbreviations: PI, Protease Inhibitors; NRTI, Nucleoside/Nucleotide Reverse Transcriptase Inhibitors; NNRTI, Non-Nucleoside Reverse Transcriptase Inhibitors; MDR, Multidrug resistance; TAMs, Thymidine Analogue-Associated Mutations. Resistance-associated codons queried (black circles) or not queried (red, open circles) by the microarray are marked. See main text for details.

a)

| Microarray | | Drug resistance mutations | | | | | | | | | | | | | | | | | | | | | |
|---|---|---|---|---|---|---|---|---|---|---|---|---|---|---|---|---|---|---|---|---|---|---|---|
| | | PI | | | | | | | | NRTI | | | | | | | | | | NNRTI | | | |
| | | | | | | | | | | Specific drug resistance | | | | | | | MDR | | | | | | |
| Region | Codon | Atazanavir +/- Ritonavir | Darunavir/Ritonavir | Fosamprenavir/Ritonavir | Indinavir/Ritonavir | Lopinavir/Ritonavir | Nelfinavir | Saquinavir/Ritonavir | Tipranavir/Ritonavir | Abacavir | Didanosine | Emtricitabine | Lamivudine | Stavudine | Tenofovir | Zidovudine | 69 insertion complex | 151 complex | TAMs | Efavirenz | Etravirine | Nevirapine | Rilpivirine |
| PR | 8 | | | | | | | | | | | | | | | | | | | | | | |
| | 30 | | | | | | ● | | | | | | | | | | | | | | | | |
| | (32) | | | | | o | | | | | | | | | | | | | | | | | |
| | 36 | | | | | | | | | | | | | | | | | | | | | | |
| | 46 | | | | ● | | | | | | | | | | | | | | | | | | |
| | (47) | | o | | | o | | | o | | | | | | | | | | | | | | |
| | 48 | | | | | | | ● | | | | | | | | | | | | | | | |
| | 50 | ● | ● | ● | | | | | | | | | | | | | | | | | | | |
| | 54 | | ● | | | | | | | | | | | | | | | | | | | | |
| | (58) | | | | | | | | o | | | | | | | | | | | | | | |
| | 71 | | | | | | | | | | | | | | | | | | | | | | |
| | (74) | | | | | | | | o | | | | | | | | | | | | | | |
| | (76) | | o | | | o | | | | | | | | | | | | | | | | | |
| | 82 | | | | ● | ● | | | ● | | | | | | | | | | | | | | |
| | (83) | | | | | | | | o | | | | | | | | | | | | | | |
| | 84 | ● | ● | ● | ● | | | | ● | | | | | | | | | | | | | | |
| | (88) | o | | | | | | | | | | | | | | | | | | | | | |
| | 90 | | | | | | ● | ● | | | | | | | | | | | | | | | |

b)

| Microarray | | Drug resistance mutations | | | | | | | | | | | | | | | | | | | | |
|---|---|---|---|---|---|---|---|---|---|---|---|---|---|---|---|---|---|---|---|---|---|---|
| | | PI | | | | | | | | NRTI | | | | | | | | | | NNRTI | | | |
| | | | | | | | | | | Specific drug resistance | | | | | | | MDR | | | | | | |
| Region | Codon | ATV +/- RTV | DNV/RTV | FAV/RTV | IDV/RTV | LPV/RTV | NFV | SQV/RTV | TPV/RTV | Abacavir | Didanosine | Emtricitabine | Lamivudine | Stavudine | Tenofovir | Zidovudine | 69 ins. complex | 151 complex | TAMs | Efavirenz | Etravirine | Nevirapine | Rilpivirine |
| RT | 41 | | | | | | | | | | | | | ● | | ● | ● | | ● | | | | |
| | 62 | | | | | | | | | | | | | | | | ● | ● | | | | | |
| | 65 | | | | | | | | | ● | ● | ● | ● | ● | ● | | | | | | | | |
| | 67 | | | | | | | | | | | | | ● | | ● | | | ● | | | | |
| | Del67 | | | | | | | | | | | | | ● | | ● | | | ● | | | | |
| | 68 | | | | | | | | | | | | | | | | | | | | | | |
| | 69 | | | | | | | | | | | | | | | | ● | | | | | | |
| | 69-70 | | | | | | | | | | | | | ● | ● | ● | ● | | ● | | | | |
| | Ins69 | | | | | | | | | | | | | | | | ● | | | | | | |
| | 70 | | | | | | | | | | | | | ● | ● | ● | ● | | ● | | | | |
| | 74 | | | | | | | | | ● | ● | | | | | | | | | | | | |
| | 75 | | | | | | | | | | | | | | | | | ● | | | | | |
| | 77 | | | | | | | | | | | | | | | | | ● | | | | | |
| | 100 | | | | | | | | | | | | | | | | | | | ● | ● | ● | ● |
| | 101 | | | | | | | | | | | | | | | | | | | ● | ● | ● | ● |
| | 103 | | | | | | | | | | | | | | | | | | | ● | | ● | |
| | 106 | | | | | | | | | | | | | | | | | | | ● | | ● | |
| | 108 | | | | | | | | | | | | | | | | | | | ● | | ● | |
| | (115) | | | | | | | | | o | | | | | | | | | | | | | |
| | 116 | | | | | | | | | | | | | | | | | ● | | | | | |
| | (138) | | | | | | | | | | | | | | | | | | | | | | o |
| | 151 | | | | | | | | | | | | | | | | | ● | | | | | |
| | 178 | | | | | | | | | | | | | | | | | | | | | | |
| | 179 | | | | | | | | | | | | | | | | | | | | | | ● |
| | 181 | | | | | | | | | | | | | | | | | | | ● | ● | ● | ● |
| | 184 | | | | | | | | | ● | | ● | ● | | | | | | | | | | |
| | 188 | | | | | | | | | | | | | | | | | | | ● | | ● | ● |
| | 190 | | | | | | | | | | | | | | | | | | | ● | | ● | |
| | 210 | | | | | | | | | | | | | ● | | ● | ● | | ● | | | | |
| | 211 | | | | | | | | | | | | | | | | | | | | | | |
| | 215 | | | | | | | | | | | | | ● | | ● | ● | | ● | | | | |
| | 219 | | | | | | | | | | | | | ● | | ● | ● | | ● | | | | |
| | (221) | | | | | | | | | | | | | | | | | | | | | | o |
| | 225 | | | | | | | | | | | | | | | | | | | ● | | | |
| | (227) | | | | | | | | | | | | | | | | | | | | | | o |
| | 230 | | | | | | | | | | | | | | | | | | | ● | | ● | ● |
| | 236 | | | | | | | | | | | | | | | | | | | | | | |
| | 238 | | | | | | | | | | | | | | | | | | | | | | |
